# Design and optimization of mucoadhesive chitosan microspheres for oesophageal delivery of fluconazole

**DOI:** 10.3389/fddev.2026.1962873

**Published:** 2026-10-01

**Authors:** H. N. Shivakumar, Ayush Parmar, Rushikesh Shinde, Sanjana S. P

**Affiliations:** 1 Centre of Innovative Science, Engineering and Education, Department of Pharmaceutics, KLE College of Pharmacy, Rajajinagar, Bengaluru, India; 2 KLE Academy of Higher Education and Research, (Deemed-to-be-University), Belagavi, India

**Keywords:** chitosan microspheres, controlled drug release, fluconazole, mucoadhesion, oesophageal candidiasis, oesophageal drug delivery, orodispersible tablet, oromucosal drug delivery

## Abstract

**Background:**

Oesophageal candidiasis remains challenging to treat owing to rapid transit of conventional oral formulations through the oesophagus, resulting in inadequate local drug delivery and reduced therapeutic efficacy. To address these limitations, the present study aimed to develop fluconazole-loaded chitosan microspheres (FCZ-CMs)-based orodispersible tablets (ODTs) that rapidly disintegrate in the oral cavity while releasing mucoadhesive microspheres capable of adhering to oesophageal mucosa, enabling sustained localized drug delivery.

**Methods:**

FCZ-CMs were developed by the emulsion cross-linking method using a 2^3^ full factorial design to optimize particle size (PS), entrapment efficiency (EE), drug release (DR), and *ex vivo* oesophageal retention (ORT). Optimised FCZ-CMs (F9) were extensively characterized by Infrared spectroscopy (FTIR), scanning electron microscopy (SEM), differential scanning calorimetry (DSC), X-ray diffractometry (XRD), and *in vitro* drug release studies. Further, the ODTs developed by direct compression of F9 were evaluated for wetting time (WT), disintegration time (DT), drug content (DC), % drug release at 60 min (%DR), similarity (*f*_*1*_ */f*_*2*_), and stability under real-time and accelerated conditions.

**Results:**

The optimised FCZ-CMs (F9) exhibited a PS (31.81 ± 1.14 μm), EE (84.12% ± 2.15%), DR (58.46% ± 1.89% at 60 min), and ORT (51.22% ± 2.11% at 30 min), demonstrating excellent suitability for localized oesophageal delivery. FTIR confirmed drug–polymer compatibility, SEM revealed spherical microspheres with a crenated surface morphology favourable for mucoadhesion, while DSC and XRD demonstrated reduced crystallinity of FCZ within the chitosan matrix. The ODTs loaded with F9 displayed high DC (99.14% ± 0.58%), short WT (19.62 ± 0.51s), and DT (23.83 ± 0.86s). The dissolution profile of ODTs remained comparable to that of F9 (*p > 0.05*), with drug release of 53.16% ± 1.24% at 60 min and similarity factor (*f*_*2*_ = 80) and difference factor (*f*_*1*_ *= 8*), confirming that the compression process did not significantly alter the release behaviour of the optimised microspheres. The formulation also remained stable under both real-time and accelerated storage conditions.

**Conclusion:**

The optimised ODTs successfully translated the performance of FCZ-CMs into a patient-friendly, oesophageal-targeted drug delivery system without compromising their controlled-release or mucoadhesive characteristics. The use of a simple, reproducible, and scalable direct compression process, together with preservation of microsphere functionality following tablet compression, highlights the translational potential of this platform for localized treatment of oesophageal candidiasis and deserves further clinical investigation.

## 1 Introduction

Oesophageal disorders represent an important group of gastrointestinal (GI) diseases that significantly affect patient health and quality of life worldwide (Shivakumar et al., 2026a). These disorders include infectious oesophagitis (Henzinger et al., 2026), gastro-oesophageal reflux disease (GERD) (Gupta et al., 2026), motility disorders (D'Angelo et al., 2026), and oesophageal malignancies (Osborne et al., 2026), many of which are associated with symptoms such as dysphagia, chest pain, reflux, chronic cough, and weight loss (Furnari et al., 2026). Among these conditions, infectious oesophagitis is particularly common in immunocompromised individuals, including patients with HIV infection, those undergoing chemotherapy, and organ transplant recipients receiving immunosuppressive therapy (Ameyaw, Korie, and Hanna, 2025). *Candida albicans* remains the most frequently identified causative pathogen of oesophageal candidiasis and is considered one of the most common opportunistic infections affecting the oesophageal mucosa. The infection leads to inflammation, ulceration, and severe discomfort during swallowing, which significantly reduces nutritional intake and deteriorates the overall quality of life of affected patients (Tamai and Kiyoura, 2025; Telbany et al., 2026). Furthermore, recurrence of the stubborn oesophageal candidiasis is frequently reported following treatment, especially in immunosuppressed populations, highlighting the need for more effective therapeutic approaches. Current therapeutic strategies for oesophageal infections primarily rely on systemic administration of antifungal agents; however, the oesophagus possesses relatively poor blood circulation compared with other gastrointestinal organs. As a result, achieving effective drug concentrations at the site of infection through systemic therapy can be challenging, often requiring higher doses that increase the risk of adverse drug reactions and systemic toxicity.

Fluconazole (FCZ) is a widely used triazole antifungal agent that exhibits potent activity against a broad spectrum of fungal pathogens, particularly *Candida* species, and is considered a first-line therapy for the treatment of oesophageal candidiasis (Takahashi et al., 2026). The chemical structure of FCZ, as depicted in Figure 1A, consists of two triazole rings attached to a fluorinated aromatic moiety, which contributes to its antifungal activity (Gandi et al., 2026). The antifungal activity of FCZ is primarily attributed to its ability to inhibit the fungal cytochrome P450-dependent enzyme lanosterol 14-α-demethylase, which plays a crucial role in the biosynthesis of ergosterol, an essential component of the fungal cell membrane. Inhibition of ergosterol synthesis disrupts membrane integrity, resulting in increased permeability and suppression of fungal growth (Bilici and Bozduman, 2026). FCZ is administered through oral or intravenous routes, with the choice of administration route depending on the patient’s clinical condition and therapeutic requirements. Oral administration is generally considered to be recommended because of the high oral bioavailability of FCZ. Despite these advantages of FCZ, delivering the drug specifically to the infected oesophageal surface appears to be quite challenging.

**FIGURE 1:**
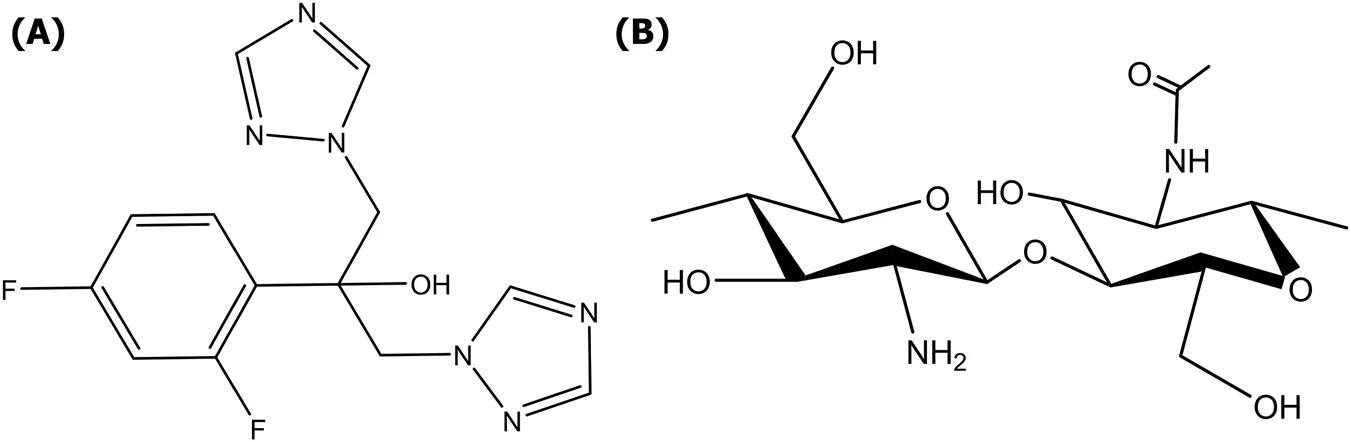
Chemical structures of Fluconazole **(A)**, a triazole antifungal compound containing two triazole rings and fluorinated aromatic groups, and Chitosan **(B)**, a linear polysaccharide composed of β-(1→4)-linked D-glucosamine units responsible for its mucoadhesive properties.

Chitosan (CS), a naturally derived polysaccharide obtained from the deacetylation of chitin, has gained considerable attention in drug delivery systems owing to its excellent biocompatibility and biodegradability (El-Naggar et al., 2022; Gieroba et al., 2026). The molecular structure of CS, illustrated in Figure 1B, is composed of β-(1→4)-linked D-glucosamine units, whose amino functional groups are responsible for its strong mucoadhesive characteristics (Shinde et al., 2026). The presence of protonable amino groups in CS enables strong electrostatic interactions with negatively charged mucin on mucosal surfaces, thereby enhancing adhesion and prolonging residence time at the site of administration (Bhattacharyya, Mukherjee, and Kaity, 2026). In addition to its mucoadhesive behaviour, CS exhibits favourable film-forming ability (Han et al., 2026), permeability-enhancing properties (Fayez et al., 2024; Mumtaz et al., 2026), and the capacity to form stable particulate systems such as microspheres (Yang et al., 2026). These characteristics make CS particularly suitable for developing localized mucosal drug delivery systems aimed at improving drug retention and therapeutic effectiveness.

The rapid oesophageal transit time appears to pose a major challenge in the development of effective localized therapies for oesophageal fungal infections. Conventional dosage forms such as tablets, capsules, and liquid preparations pass quickly through the oesophagus and reach the stomach, limiting the opportunity for drug absorption or localized therapeutic action at the site of infection. Consequently, there is growing interest in designing drug delivery systems capable of prolonging the residence time of drugs on the oesophageal surface to ensure sustained contact with the mucosal surface. Among the various approaches explored, bioadhesive microparticulate systems have emerged as promising candidates for achieving localized oesophageal targeting (Manna et al., 2026). These systems, such as pellets, beads, or granules, distribute uniformly over the mucosal surface, thereby increasing the surface area for drug release and enhancing drug retention at the target site. Additionally, microparticulates can be engineered using suitable polymers to provide mucoadhesive properties and site-specific drug release, allowing prolonged exposure of the drug to the oesophageal mucosa while minimizing systemic side effects (Sapra et al., 2024). Considering these advantages, the development of FCZ-loaded CS microspheres (FCZ-CMs) offers a promising strategy for improving the treatment of oesophageal candidiasis. Microparticulates can enhance local drug availability by prolonging residence time within the oesophagus and ensuring sustained antifungal activity directly at the site of infection.

We hypothesized that the integration of FCZ-CMs into an orodispersible tablet (FCZ-CM-loaded ODTs) would provide a novel platform for localized oesophageal drug delivery by rapidly disintegrating in the oral cavity, which could enable easy deposition of mucoadhesive microspheres onto the oesophageal mucosa, thereby prolonging local residence and enabling sustained therapeutic efficacy. It was further hypothesized that systematic optimization using a Design of Experiments (DoE) approach would yield a robust microsphere formulation with optimal particle size (PS), entrapment efficiency (EE), oesophageal retention (ORT), and controlled drug release (DR), while subsequent transformation into an orodispersible tablet would preserve these critical quality attributes. To the best of our knowledge, no previous study has reported fluconazole-loaded chitosan microsphere-based ODTs specifically designed for localized oesophageal delivery. The proposed formulation is therefore expected to represent a reproducible and scalable drug delivery platform with the potential to improve the localized management of oesophageal candidiasis while minimizing unnecessary systemic drug exposure.

## 2 Materials and methods

### 2.1 Materials

Fluconazole (FCZ) I.P. was procured from Vapi Care Pharmaceuticals, Vapi, India. Chitosan (CS; medium molecular weight, 200 kDa; degree of deacetylation, 75%–85%) was obtained from Rajesh Chemicals, Mumbai, India. Liquid paraffin was sourced from Qualigens Fine Chemicals, India. Glutaraldehyde was purchased from Merck, Mumbai, India. Docusate sodium was supplied by Sigma-Aldrich, Mumbai, India. Potassium dihydrogen phosphate and di-potassium hydrogen phosphate were obtained from Loba Chemical Pvt. Ltd., Mumbai, India. Potassium hydroxide, potassium chloride, and magnesium chloride were procured from S.D. Fine Chemicals, Mumbai, India. Disodium sulphate and sodium chloride were also obtained from Loba Chemical Pvt. Ltd., Mumbai, India. Additionally, acetic acid, hexane, and petroleum ether were acquired from Central Drug House Pvt. Ltd., Gujarat, India. All reagents and chemicals used were of analytical or laboratory reagent grade.

### 2.2 Design of experiment

Design-Expert® software (version 13; Stat-Ease Inc., Minneapolis, United States of America) was employed to set up the experimental design. The 2^3^-full factorial design was employed to study the effect of independent variables on particle Size (PS), entrapment efficiency (EE), and *in vitro* percentage drug release at the end of 60 min (Q_60_), and *ex vivo* percentage oesophageal retention of particles at the end of 30 min (ORT_30_). The independent variables, along with the coded and actual values, are represented in Table 1, while the associated eight experimental batches designed are depicted in Table 2.

**TABLE 1:** Variables used for the optimisation of fluconazole-loaded microspheres.

Factors	Coded levels	
Independent variables	Low (−1)	High (+1)
X1: D:P ratio^a^	1:1	1:2
X2: Polymer conc. (%)	1.0	2.0
X3: Stirring speed (rpm)	1,500	2,500
Dependent variables	Constraints	
R1: PS^b^ (µm)	Minimize	
R2: EE^c^ (%)	Maximize	
R3: Q_60_ ^d^ (%)	Maximize	
R4: ORT_30_ ^e^ (%)	Maximize	

^a^ Drug-to-Polymer Ratio.

^b^ Particle size.

^c^ Entrapment Efficiency.

^d^ In vitro percentage drug release at the end of 60 min.

^e^ Ex vivo percentage retention of particles at the end of 30 min.

**TABLE 2:** Formulation batches using the 2^3^-full factorial design.

Batches	Factor X_1_	Factor X_2_	Factor X_3_
	D:P ratio	Polymer conc. (%)	Stirring speed (rpm)
F1	1:1	1	1,500
F2	1:2	1	2,500
F3	1:2	2	2,500
F4	1:2	2	1,500
F5	1:1	1	2,500
F6	1:1	2	1,500
F7	1:1	2	2,500
F8	1:2	1	1,500

### 2.3 Preparation of fluconazole-loaded microspheres

FCZ-CMs were formulated using the emulsion cross-linking method, as described by Thanoo et al., 1992; Thanoo, Sunny, and Jayakrishnan, 1992). CS was employed as the bioadhesive polymeric carrier, with glutaraldehyde acting as the cross-linking agent. Initially, CS was dissolved in a 2% v/v aqueous solution of acetic acid. FC was dissolved with a minimal amount of ethanol to ensure complete solubilization. The drug solution was added to the polymeric solution to obtain a clear aqueous phase. The resulting homogeneous aqueous phase was extruded dropwise through a No. 20 syringe needle into 100 mL of a liquid paraffin mixture (heavy and light in a 1:1 ratio) containing 0.2% w/v docusate sodium, under continuous stirring using a propeller stirrer (RO-127/A, Remi Elektrotechnik Ltd., India) at room temperature. The emulsion was stirred continuously for 3 h to ensure complete removal of the aqueous phase, resulting in the formation and stabilization of microspheres. After 150 min of stirring, 2.5 mL of toluene pre-saturated with glutaraldehyde was added to initiate cross-linking (Kuang et al., 2026). Subsequently, the microspheres were collected by filtration, washed repeatedly with hexane to eliminate residual oil, followed by extensive washing with ice-cold water to remove residual solvents. Finally, the microspheres were dried in a hot air oven at 50 ° C for 20 min And stored in a desiccator at room temperature until further use (Figure 2).

**FIGURE 2:**
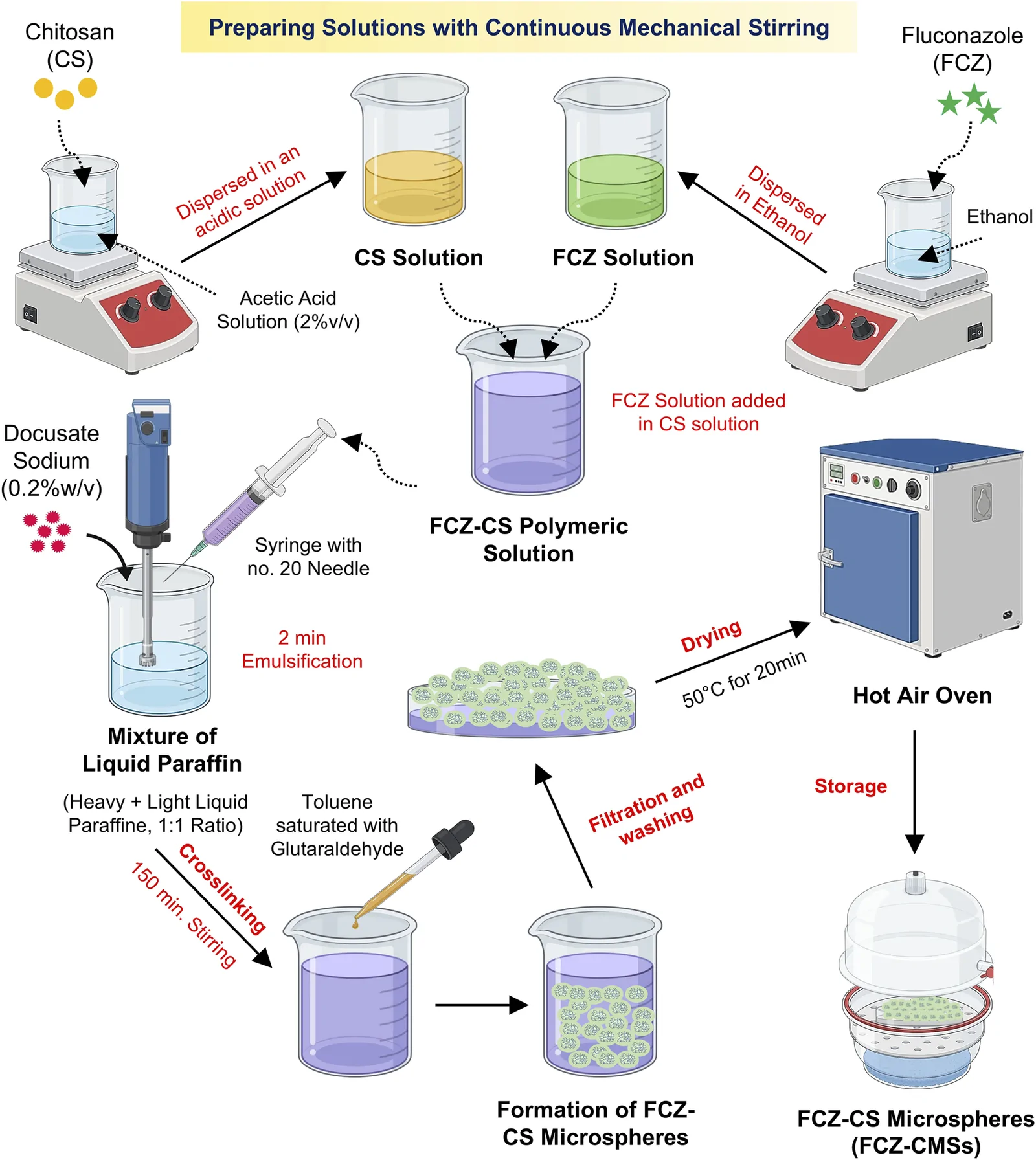
Schematic illustration of preparation of fluconazole-loaded chitosan microspheres by emulsion cross-linking, involving polymer dissolution, drug incorporation, emulsification in liquid paraffin, glutaraldehyde-mediated cross-linking, followed by washing, drying, and collection of microspheres.

### 2.4 Optimization of dependent variables

A 2^3^ full factorial design was employed and analyzed using Design-Expert® software version 13 (Stat-Ease Inc., United States of America) to facilitate the development and optimization of FCZ-CMs. Constraints were set on the dependent variables that included PS (µg), EE (%), Q_60_ (%), and ORT_30_ (%) to optimize the independent variables.

#### 2.4.1 Particle size

The PS of the prepared FCZ-CMs was determined using optical microscopy (Vision 2000; Labomed, India). An optical microscope equipped with an ocular micrometer and a stage micrometer was used for the analysis. Before measurement, the eyepiece micrometer was calibrated using the stage micrometer to ensure accuracy. A small quantity of microspheres from each batch was spread on a clean glass slide and viewed under the microscope. The diameters of more than 400 microspheres were measured by moving the stage of the microscope in a zigzag manner to obtain statistically reliable data. The frequency distribution of PS was then plotted to evaluate the size distribution, from which the average PS for each batch was calculated. The average mean diameter (dIn) and volume surface (dvs) were determined using Edmondson’s Equations 1 and 2.

$$d_{In}=\frac{\sum nd}{\sum n}$$ (1)

$$d_{vs}=\frac{\sum {nd}^{3}}{\sum {nd}^{2}}$$ (2)

Where ‘n’ is the number of microspheres observed, ‘d’ is the mean size range, and ‘N’ is the number of particles per unit mass (Avlani et al., 2023).

#### 2.4.2 Entrapment efficiency and drug loading

An accurately weighed amount of FCZ-CMs was transferred into 25 mL of artificial saliva (pH 7.4) to determine the drug content (DC) in the CMs. The dispersion obtained was then placed on a mechanical shaker (2 ML Magnetic Stirrer, REMI Equipment Ltd., India) and agitated continuously for 24 h to ensure complete drug recovery. After the shaking period, the dispersion was filtered, and the resulting filtrate was analyzed using a UV-Visible spectrophotometer (Shimadzu 1900i UV–Visible Spectrophotometer, Japan), using artificial saliva as the blank. The drug loading (DL) and entrapment efficiency (EE) were calculated according to Equations 3 and 4 **(** Avlani et al., 2023; Tekade et al., 2026 **)**.

$$\text{DL }\left(\%\right)=\frac{\text{Amount}\;\text{of}\;\text{drug}\;\text{in}\;\text{microspheres}\;\left(\text{mg}\right)}{\text{Amount}\;\text{of}\;\text{microspheres}\;\left(\text{mg}\right)}\times 100$$ (3)

$$\text{EE }\left(\%\right)=\frac{\%\;\text{Drug}\;\text{loading}}{\%\;\text{Theoretical}\;\text{loading}\;}\times 100$$ (4)

#### 2.4.3 *In Vitro* drug release studies

FCZ-loaded microspheres (equivalent weight 50 mg FCZ) were subjected to *in vitro* dissolution testing using a Type IV flow-through dissolution apparatus (Model No. EFT- 01, Electrolab India Pvt. Ltd., India). The dissolution medium consisted of freshly prepared (60 mL) artificial saliva (pH 7.4), maintained at a flow rate of 1 mL/min. At each sampling interval, a predetermined volume of the dissolution medium was withdrawn and immediately replaced with an equal volume of fresh artificial saliva (pH 7.4). The study was conducted for a total duration of 60 min, during which samples were withdrawn at 10 min Time intervals. The collected samples were then analyzed spectrophotometrically to determine the amount of drug released. The dissolution experiments were performed in triplicate to ensure reproducibility and statistical reliability of the results (Muta, Koppisetti, and Garg, 2026).

#### 2.4.4 *Ex vivo* oesophageal retention studies

The mucoadhesive potential of the FCZ-CMs was assessed using a modified USP disintegration apparatus. Fresh goat oesophageal mucosa (approximately 5 × 1 cm), obtained from a local slaughterhouse, was carefully excised and used as the biological substrate. The mucosal tissue was affixed onto a glass slide using a suitable adhesive to ensure stability during the experiment. A known quantity of microspheres (approximately 100 particles) was evenly distributed over the surface of the mounted mucosa. The prepared slide was then attached to the arm of the disintegration apparatus and immersed in artificial saliva (pH 7.4), maintained at 37 ° C ± 0.5 ° C to simulate physiological conditions. The apparatus was operated to produce a controlled, periodic up-and-down motion, providing a standardized mechanical challenge for comparative assessment of microsphere retention. At specific time intervals over a duration of 60 min, the apparatus was temporarily halted, and the number of microspheres remaining adhered to the mucosal surface was determined using microscopic examination. All measurements were performed in triplicate to ensure reproducibility. The percentage mucoadhesion was subsequently calculated using Equation 5 (Avlani et al., 2023).

$$\text{Mucoadhesion }\left(\%\right)=\frac{\text{Number}\;\text{of}\;\text{microspheres}\;\text{adhered}}{\text{Initial}\;\text{numbers}\;\text{of}\;\text{microspheres}}\times 100$$ (5)

### 2.5 Evaluation of fluconazole-loaded microspheres

#### 2.5.1 Fourier transform infrared (FTIR) spectroscopy

FTIR spectra of pure FCZ, CS, physical mixture, and optimised FCZ-CMs were recorded using a Jasco FTIR-460PLus spectrometer (Tokyo, Japan). Before analysis, the samples were thoroughly blended with potassium bromide (KBr) powder and loaded into a diffuse reflectance sampling accessory before exposure to infrared radiation. Spectral acquisition was performed over a wavenumber range of 400–4,000 cm^-1^ with a KBr blank run-in advance to establish baseline characteristics and identify the distinctive absorption peaks of the FCZ. The resulting spectra were examined to detect the presence and retention of characteristic FCZ peaks in the physical mixture and formulation (Shivakumar et al., 2026b).

#### 2.5.2 Morphological evaluation

The morphology and surface characteristics of the prepared optimised FCZ-CMs were examined using SEM (JSM-840A, JEOL Ltd., Japan). For sample preparation, a small amount of microsphere powder was gently sprinkled onto a double-sided adhesive tape fixed on an aluminum stub. The stubs were then coated with a thin layer of gold (approx. 30 nm) using a JEOL Fine Coat JFC 1100 E sputter coater under high vacuum and high voltage conditions. The gold-coated samples were subsequently analyzed under a 20 kV electron beam to obtain high-resolution images of the microspheres, which provided insights into their shape, surface texture, and structural uniformity (Wali et al., 2025).

#### 2.5.3 Differential scanning calorimetry (DSC)

Thermal characterization was carried out using a differential scanning calorimeter (DSC-60, Shimadzu, Kyoto, Japan). The samples were accurately weighed, sealed in aluminum pans, and heated at a constant rate of 10 ° C/min under a nitrogen atmosphere with a flow rate maintained between 80 and 100 mL/min. Considering the melting behaviour of FCZ, thermograms were recorded over a temperature range of 50 ° C–250 ° C. The degree of crystallinity (Xc) of the physical mixture and formulation was determined relative to FCZ as the reference standard using Equation 6 (Nanjappa et al., 2026a).

$$\text{Xc}=\frac{\Delta \text{Hm}}{\left(1\;‐\;w\right).\Delta H{}^{\circ}m\;}\times 100$$ (6)

Where ‘ΔH^0^m’ is the heat of fusion for 100% crystalline FCZ, ‘ΔHm’ is the measured heat of fusion, and ‘w’ is the weight fraction of the sample within the carrier matrix.

#### 2.5.4 Crystallinity assessment

An X-Ray Diffractometer (Rigaku Miniflex 600, Tokyo, Japan) was used for the analysis of samples for crystallinity. The diffractogram of the FCZ, CS, Physical Mixture, and optimised FCZ-CMs was acquired at a scanning speed of 0.5 s per step, operating at 40 kV and 40 mA, and utilizing a Cu radiation source. The degree of crystallinity (DoC) of the samples relative to FCZ was calculated using Equation 7 (Nanjappa et al., 2026b).

$$\text{DoC}=\frac{I_{Sam}}{I_{ref}}\times 100$$ (7)

Where ‘**Isam**’ denotes the sample’s highest characteristic peak, and ‘**Iref**’ refers to the peak height at the same angle in the reference, measured by intensity.

#### 2.5.5 *In Vitro* drug release kinetics

Attempts were made to fit the dissolution data of the FCZ-CMs into a first-order model (Equation 8) to determine the drug release kinetics. This analysis aids in evaluating the drug release kinetics and comparing the release behaviour among various batches of formulations.

$$Q_{t}=Q_{0}e^{-k_{1}t}$$ (8)

Where **‘Q**_**0**_ **’** and **‘Q**_**t**_ **’** indicate the drug release levels at specific time intervals, 0 and at a time ‘**t**’, respectively, ‘**K1**’ is the first-order rate constant (Nyamba et al., 2024).

The dissolution data were analyzed by fitting into various mathematical models, including the Higuchi diffusion model (Equation 9) and the Korsmeyer-Peppas model (Equation 10), to understand and characterize the underlying mechanism of drug release.

$$Q_{t}=K_{H}\sqrt{t}$$ (9)

$$\frac{Q_{t}}{Q_{\infty }}=K_{p}t^{n}$$ (10)

Where ‘**Qt**’ is FCZ release amount at time ‘**t**’, ‘**K**_**H**_’ is the Higuchi release constant reflecting the rate of diffusion, ‘**Q∞**’ is the maximum amount of drug released achievable over an infinite period, ‘**K**_**p**_’ is the formulation-dependent release rate constant, and ‘**n**’ is the release exponent used to characterize the underlying drug release mechanism (Nanjappa et al., 2026a).

### 2.6 Formulation of FCZ-CMs-loaded orodispersible tablets

FCZ-CMs-loaded orodispersible tablets (ODTs) were prepared by the direct compression technique using the optimised FCZ-CMs. The quantitative composition of the ODT is presented in Table 3. An appropriate quantity of optimised FCZ-CMs (F9), together with the remaining excipients, including directly compressible diluents, superdisintegrants, sweetening agent, lubricant, and glidant, was weighed accurately. Before blending, all formulation components were passed through a #44 mesh sieve to ensure uniform PS and to remove any aggregates. The FCZ-CMs, along with the excipients, were transferred to a polyethene bag and blended thoroughly to obtain a homogeneous powder mixture. Magnesium stearate and talc were incorporated as the final blending step and mixed gently for 2–3 min to achieve uniform distribution while minimising the risk of over-lubrication. The resulting powder blend was compressed using a rotary tablet compression machine (Model RSB-4, Rimek Mini press, IRM Enterprises Pvt. Ltd., India) equipped with 8 mm flat-faced punches. The compression force was optimised to produce ODTs with adequate mechanical strength (approximately 3–4 kg/cm^2^) to ensure rapid disintegration. The prepared tablets were stored in airtight containers under ambient conditions until further physicochemical and performance evaluation (Alhamhoom et al., 2025).

**TABLE 3:** Composition of the optimised Fluconazole-loaded chitosan microsphere-based orodispersible tablet.

Ingredients	FCZ-CMs ODT
FCZ-loaded microspheres (equivalent to 50 mg)	100 mg
Microcrystalline cellulose	25 mg
Mannitol	25 mg
Cros-povidone	5 mg
Croscarmellose sodium	5 mg
Total tablet weight	160 mg

### 2.7 Characterization of FCZ-CMs-loaded orodispersible tablets

#### 2.7.1 Pre-compression characterisation of the powder blend

The powder blend comprising FCZ-CMs and excipients was evaluated for micromeritic properties before compression to assess flowability and compressibility. Bulk density, tapped density, angle of repose, Carr’s compressibility index, and Hausner ratio were determined according to standard pharmacopoeial procedures.

##### 2.7.1.1 Bulk density

An accurately weighed quantity of the powder blend was gently introduced into a clean, dry 10 mL graduated measuring cylinder without applying external force. The cylinder was allowed to stand until the powder bed stabilised, and the initial volume occupied by the powder was recorded. Measurements were carried out in triplicate, and the results are reported as mean ± SD. Bulk density (B.D.) was calculated using Equation 11 (Alam et al., 2024).

$$\begin{array}{cccc} & B.\;D.\;\left({g/\text{cm}}^{3}\right)=\frac{\text{Weight}\;\text{of}\;\text{powder}\;\left(g\right)}{\text{Bulk}\;\text{volume}\;\left(\text{mL}\right)} & & \end{array}$$ (11)

##### 2.7.1.2 Tapped density

Following the bulk density measurement, the same graduated measuring cylinder containing the powder blend was secured in a tapped density tester (Electrolab ETD-1020, Electrolab India Pvt. Ltd., Mumbai, India). The cylinder was tapped at a constant rate until no further change in powder volume was observed (Alam et al., 2024). The final compacted volume was recorded, and the tapped density (T. D.) was calculated using Equation 12. Measurements were carried out in triplicate, and the results are reported as mean ± SD.

$$\begin{array}{cccc} & T.D.\;\left({g/\text{cm}}^{3}\right)=\frac{\text{Weight}\;\text{of}\;\text{powder}\;\left(g\right)}{\text{Tapped}\;\text{volume}\;\left(\text{mL}\right)} & & \end{array}$$ (12)

##### 2.7.1.3 Carr’s compressibility index

The compressibility characteristics of the powder blend were evaluated using Carr’s Compressibility Index (C.I.) (Alhamhoom et al., 2025), which was calculated from the measured bulk and tapped density values according to Equation 13. All determinations were performed in triplicate and are expressed as mean ± SD.

$$\begin{array}{cccc} & C.I.\;\left(\%\right)=\frac{\text{Tapped}\;\text{Density}-\text{Bulk}\;\text{Density}}{\text{Tapped}\;\text{Density}}\times 100 & & \end{array}$$ (13)

##### 2.7.1.4 Hausner ratio

The Hausner ratio (H.R.) was calculated using the corresponding bulk and tapped density values as shown in Equation 14. The obtained values were used to assess the flow characteristics of the powder blend (Alhamhoom et al., 2025). Measurements were performed in triplicate, and the results are presented as mean ± SD.

$$\begin{array}{cccc} & H.R.=\frac{\text{Tapped}\;\text{Density}}{\text{Bulk}\;\text{Density}}\times 100 & & \end{array}$$ (14)

##### 2.7.1.5 Angle of repose

The angle of repose $\left(\theta \right)$ was determined using the fixed-funnel method. A funnel was positioned at a fixed height above a horizontal surface, and the powder blend was allowed to flow freely through the funnel to form a conical pile. After the powder flow had ceased, the height of the cone and the radius of its base were measured. The angle of repose was calculated using Equation 15 (Müller, Fimbinger, and Brand, 2021). Each determination was performed in triplicate, and the results are expressed as mean ± SD.

$$\begin{array}{cccc} & \theta =\tan^{-1}\left(\frac{h}{r}\right)\times 100 & & \end{array}$$ (15)

Where ‘**h’** represents the height of the pile of powder cone and ‘**r’** denotes the radius of its base.

#### 2.7.2 Post-compression characterisation of the orodispersible tablet

The prepared FCZ-CMs-loaded ODTs were evaluated for post-compression characteristics, including weight variation (WV), tablet thickness, hardness, friability, wetting time (WT), *in vitro* disintegration time (DT), DC, and *in vitro* dissolution. All measurements were performed according to pharmacopoeial procedures, and the results are expressed as mean ± standard deviation (SD).

##### 2.7.2.1 Weight variation

Twenty tablets were randomly selected and weighed individually using an analytical balance (Shimadzu BL-220H, Kyoto, Japan). The percentage deviation of individual tablet weights from the mean weight was calculated according to the Indian Pharmacopoeia (IP) (Alhamhoom et al., 2024).

##### 2.7.2.2 Hardness

The crushing strength of the tablets was determined using a Monsanto hardness tester (Monsanto, United States of America). Ten tablets from each formulation were analysed individually, and the hardness values were reported in kg/cm^2^ (Alhamhoom et al., 2024).

##### 2.7.2.3 Thickness

Tablet thickness was measured with a digital Vernier calliper (Mitutoyo Corporation, Tokyo, Japan). Measurements were obtained from ten tablets selected at random, and the average value was calculated to evaluate dimensional uniformity (Avlani et al., 2023).

##### 2.7.2.4 Friability

Friability was determined using a Roche friabilator (Electrolab EF-20, Electrolab India Pvt. Ltd., Mumbai, India). Tablets with a total weight of approximately 6.5 g were accurately weighed (W_0_) and placed in the friabilator. The instrument was operated at 25 rpm for 4 min (100 revolutions). Upon completion of the test, the tablets were removed, dedusted to eliminate loose particles, and reweighed (W_1_). The percentage friability was calculated using Equation 16 (Nanjappa et al., 2026b). A friability value below 1% was considered acceptable.

$$Friability\;\left(\%\right)=\frac{W_{o}-W_{1}}{W_{o}}\times 100$$ (16)

##### 2.7.2.5 Wetting time

Wetting time was determined by placing an individual tablet in a Petri dish containing 10 mL of artificial saliva (pH 7.4) maintained at 37 ° C ± 0.5 ° C. The tablet was allowed to absorb water under static conditions, and the time required for complete wetting, indicated by uniform water penetration throughout the tablet without any visible dry regions, was recorded using a digital stopwatch (Mohamed et al., 2026).

##### 2.7.2.6 *In vitro* disintegration time

The *in vitro* disintegration time of the prepared ODTs was determined using a USP disintegration test apparatus (Electrolab ED-2L, Electrolab India Pvt. Ltd., Mumbai, India). Six tablets from each formulation batch were placed individually into the six tubes of the apparatus, each fitted with a standard plastic disc. Distilled water maintained at 37 ° C ± 0.5 ° C was used as the disintegration medium. The apparatus was operated according to the USP procedure, and the time required for each tablet to disintegrate completely without any palpable residue was recorded using a digital stopwatch. The average DT was calculated from six determinations (Brniak et al., 2015; Raeisi Estabragh, Pardakhty, and Pardakhty, 2025).

##### 2.7.2.7 Drug content

Ten tablets were finely powdered, and an amount equivalent to the labelled FCZ content was transferred into a volumetric flask containing the analytical solvent. The dispersion was sonicated for 15 min, filtered through Whatman No. 1 filter paper, appropriately diluted, and analysed by UV-visible spectrophotometry. Drug content (DC) was determined using the corresponding calibration curve (Brniak et al., 2015).

##### 2.7.2.8 *In Vitro* dissolution study

The dissolution behaviour of the FCZ-CMs-loaded ODTs was evaluated using a Type IV flow-through dissolution apparatus. Dissolution studies were carried out under the same experimental conditions employed for the microsphere formulation. At predetermined time intervals, aliquots (5 mL) were withdrawn and immediately replaced with an equal volume of fresh dissolution medium maintained at the same temperature. Samples were filtered, analysed spectrophotometrically, and the cumulative percentage drug release was calculated. The *in vitro* dissolution profiles were constructed by plotting the cumulative percentage of drug released against time. The similarity factor (***f***_***2***_) and dissimilarity factor (***f***_***1***_) were calculated using Equations 17 and 18, respectively, to compare the dissolution profiles of FCZ-CMs-loaded ODTs with the optimised FCZ-CMs (F9) (Alhamhoom et al., 2025).

$$f_{1=}\frac{\left\{\left\{\sum_{t=1}^{n}\left|Rt-Tt\right|\right\}\right\}}{\sum_{t=1}^{n}Rt}\times 100$$ (17)

$$f_{2}=50\times \log\left[{\left\{1+\frac{1}{n}\sum_{r=1}^{n}Wt\left(Rt-Tt\right)\right\}}^{-0.5}\times 100\right]$$ (18)

Where ‘**n**’ denotes the total number of sampling time points, ‘***Rt***’ represents the mean percentage of drug released from the reference formulation at time ‘***t***’; ‘***Tt***’ represents the corresponding mean percentage of drug released from the test formulation; and ‘***Wt***’ denotes the weighting factor, which was assigned a value of 1.

### 2.8 Stability study

The FCZ-CMs-loaded ODTs were subjected to stability studies over 3 months under real-time conditions of 25 ° C ± 2 ° C with 60% ± 5% relative humidity (RH) and 40 ° C ± 2 ° C with 75% ± 5% RH for accelerated conditions (ICH, 2003). At the end of the study, critical parameters such as weight variation, friability, hardness, disintegration time, and drug content were evaluated. The results were then compared with the initial data recorded before the stability testing.

### 2.9 Statistical analysis

The experimental design was set up, and the results obtained were statistically analysed using Design-Expert® software (Version 13.0.5.0, Stat-Ease Inc., Minneapolis, MN, United States of America). A *p*-value of less than 0.05 was considered statistically significant. For Pareto analysis, the Bonferroni limit generated by Design-Expert® was used as a conservative threshold for screening and interpreting the standardized effects of the formulation and process variables. Origin 8.5 (Origin Lab Corporation, 433 Northampton, MA, United States of America) and GraphPad Prism 8.0.2 (GraphPad Software, LLC, San Diego, 434 C A, United States of America) were also used for graphical presentation and comparison of raw data generated.

## 3 Results and discussion

FCZ is a concentration-dependent antifungal agent, and therefore maintaining therapeutic FCZ concentrations at the site of infection is vital for achieving effective clinical outcomes. Sub-therapeutic local concentrations may result in inadequate fungal suppression and could potentially contribute to treatment failure or development of antifungal resistance. Therefore, the controlled release of FCZ from the FCZ-CMs is vital for maintaining sufficient local FCZ exposure at the oesophageal mucosa (Abd El Gawad and Shady, 2026). On the other hand, conventional topical applications rapidly transit through the oesophagus, typically within a few seconds, which limits drug exposure to the oesophageal mucosa, affecting therapeutic efficiency. The development of a micro-particulate system capable of adhering to the mucosal surface and releasing the drug to the site-specific could, therefore, substantially improve localized antifungal therapy. FCZ-CMs are an effective localized drug delivery platform for the treatment of oesophageal candidiasis by enhancing the residence time of the drug at the site of infection. These systems are likely to enhance drug exposure at the oesophageal surface, prolong drug retention, and may potentially reduce unnecessary systemic exposure commonly associated with conventional systemic antifungal therapy.

In the present study, FCZ-CMs were prepared using CS to achieve prolonged oesophageal retention and site-specific drug release. Different batches were developed by varying the polymer concentration and drug-to-polymer ratios based on preliminary optimization studies. The microspheres were produced using an appropriate solvent evaporation technique to ensure uniform drug distribution within the polymeric matrix. CS was used as a polymer considering its mucoadhesive and site-specific release properties, which can promote interaction with the oesophageal mucosa and enhance the residence time of the microparticulate system (Deore et al., 2026; Tekade et al., 2025). Considering the good solubility of FCZ (∼5.5 mg/mL), the W/O emulsification method was employed to produce FCZ-CMs. During the preparation process, the FCZ and CS were dissolved in an aqueous medium to obtain a homogeneous solution before microsphere formation. The continuous phase of liquid paraffin is likely to improve the EE of CMSs by avoiding drug dissipation (More et al., 2026). Dioctyl sodium sulphosuccinate is an anionic surfactant used as an emulsifier that favours the formation of a W/O type of emulsion. Subsequent solvent removal enabled the formation of solid microspheres with the drug entrapped within the polymeric matrix. The prepared microspheres were then subjected to further physicochemical characterization.

### 3.1 Design of experiment (DoE)

A 2^3^ full factorial design was employed to evaluate the influence of process variables on the key attributes of FCZ-CMs. Three independent variables were selected based on preliminary screening studies, including drug-to-polymer ratio (X1), polymer concentration (X2), and stirring speed (X3). Each factor was investigated at two levels, resulting in eight experimental runs (F1–F8). The prepared CMs were evaluated for multiple responses, including PS (µm), EE (%), Q_60_ (%), and ORT_30_(%). The experimental responses obtained for all formulations are summarized in Table 4.

**TABLE 4:** Physicochemical characterization of different prepared fluconazole-loaded microsphere batches.

Runs	PS^a^ (µm)	V_SM_ ^b^ (µm)	EE^c^ (%)	Q_60_ ^d^ (%)	ORT_30_ ^e^ (%)
F1	40.66 ± 2.18	51.89 ± 1.11	76.23 ± 0.88	55.71 ± 1.42	46.64 ± 1.34
F2	36.71 ± 1.01	54.22 ± 2.30	84.95 ± 1.84	58.18 ± 2.10	50.32 ± 2.13
F3	65.12 ± 3.20	74.49 ± 1.89	90.85 ± 0.54	46.13 ± 2.75	35.68 ± 1.78
F4	89.49 ± 2.23	97.43 ± 2.03	87.86 ± 1.10	38.63 ± 1.25	32.64 ± 1.25
F5	34.64 ± 1.03	51.36 ± 0.88	77.63 ± 1.24	62.41 ± 1.48	62.16 ± 1.18
F6	85.76 ± 3.48	88.86 ± 1.15	80.67 ± 1.10	40.93 ± 3.05	36.28 ± 2.16
F7	45.33 ± 1.25	59.03 ± 2.10	81.77 ± 0.35	52.56 ± 2.31	44.24 ± 1.31
F8	44.96 ± 1.34	56.25 ± 1.20	83.94 ± 1.45	50.53 ± 2.71	44.34 ± 1.02

^a^ Particle size.

^b^ Volume surface mean.

^c^ Entrapment efficiency.

^d^ In vitro percentage drug release at the end of 60 min; and.

^e^ Ex vivo percentage oesophageal retention of particles at the end of 30 min *All data are expressed as mean ± SD (n = 3).

Statistical analysis was performed to determine the significance of each processing variable in order to arrive at the optimised formulation. Regression analysis was performed to determine the quantitative influence of selected formulation variables on the responses. The regression models generated for each response revealed the direction and magnitude of individual factors and their interactions. It helped to identify the most critical factors that significantly impact each response, facilitating the optimization process. The adequacy of the developed regression models was further assessed using residual diagnostic plots. Normal probability plots of the externally studentized residuals were examined for PS (R1), EE (R2), Q_60_ (R3), and ORT_30_ (R4). The residuals showed an approximately linear distribution along the theoretical normal probability line, with no pronounced deviations indicative of substantial non-normality, supporting the reasonable adequacy of the normality assumption for the fitted models.

#### 3.1.1 Impact on particle size (R1)

PS is a critical quality attribute of microparticulate drug delivery systems, as it significantly influences drug release behaviour, surface area, mucoadhesion, and retention at the target site. In the present study, the PS of the developed FCZ-CMs ranged from 34.64 ± 1.03 to 89.49 ± 2.23 µm (Table 4). The variation observed among the formulations indicates that the processing variables played a significant role in controlling the PS of the microspheres. The influence of the independent variables on PS was further illustrated by the Pareto chart (Figure 3A), which highlights the relative magnitude and direction of factor effects. The Bonferroni limit of 4.38175 indicated in the Pareto plot represents the conservative threshold generated by DoE for screening the standardized effects. Wherein, effects exceeding this limit were included in the model construction. The mathematical model generated for PS is expressed by Equation 19.

$$\text{PS }\left(R1\right)=55.33+16.09.X2-9.88.X3$$ (19)

**FIGURE 3:**
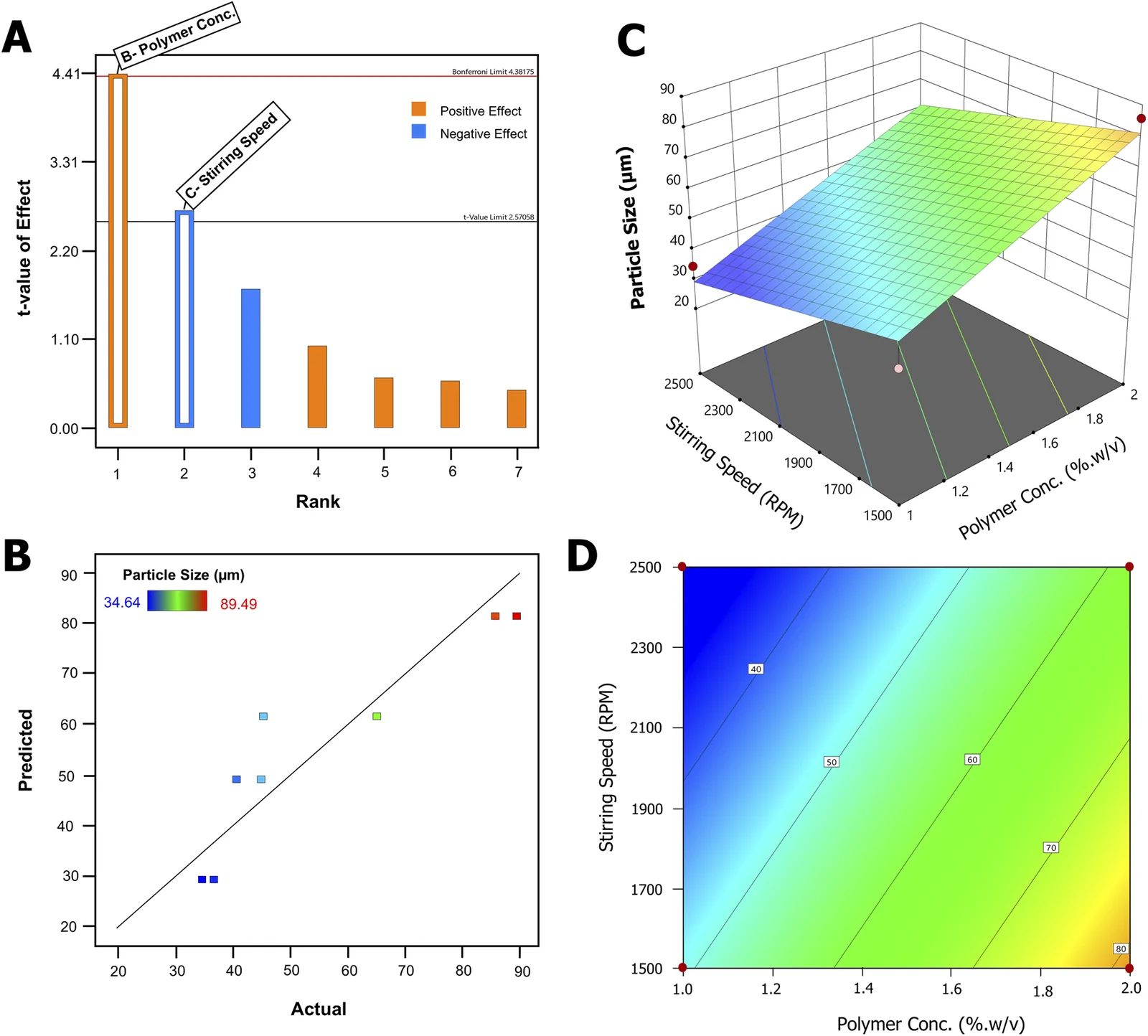
Pareto plot **(A)**, correlation plot comparing actual and predicted values **(B)**, three-dimensional response surface plot **(C)**, and two-dimensional contour plot **(D)** illustrating the effects of stirring speed and polymer concentration on particle size of fluconazole-loaded microspheres. The color gradient represents the magnitude of the response (blue: lower and red: higher particle size).

The regression model demonstrated statistical significance with a model F-value of 13.37 (*p* < 0.05), confirming that the model adequately describes the relationship between the selected variables and the response. The adjusted *R* ^2^ value of 0.7795 and predicted *R* ^2^ value of 0.5968 indicate reasonable agreement between the predicted and experimental values. This indicates good agreement between experimental and predicted PS values. The close distribution of data points along the diagonal line indicates that the model is able to accurately predict the response within the studied experimental region (Figure 3B). Furthermore, the adequate precision value of 8.21, which is greater than the desirable value of 4, confirms that the model has an adequate signal and can be used to navigate the design space.

The Pareto chart indicates that polymer concentration (X2) and stirring speed (X3) significantly influenced PS, whereas the drug-to-polymer ratio displayed a comparatively lesser effect. Statistical analysis from ANOVA further confirmed that polymer concentration and stirring speed had a significant effect on PS (*p < 0.05*), while the influence of drug-to-polymer ratio was statistically insignificant. Polymer concentration exhibited a positive effect, suggesting that increasing the polymer concentration resulted in larger PS. This behaviour can be attributed to the increased viscosity of the polymer solution with an increase in polymer concentration, which reduces shear-induced droplet breakup during emulsification, thereby producing larger particles. A similar effect of an increase in PS of CMs with an increase in CS concentration has been recently reported (Şentürk et al., 2026; Sanjana et al., 2026). Conversely, stirring speed demonstrated a negative effect on PS, indicating that higher stirring speeds resulted in smaller microspheres. Increased agitation enhances shear forces during the emulsification process, promoting the formation of smaller droplets that subsequently solidify into smaller microspheres (Zhang et al., 2026). It is well reported in the literature that a reduction in PS enhances mucosal permeation by increasing the available surface area for interaction with the epithelium, thereby improving drug solubility, dissolution rate, and permeability across the mucosal barrier (Tekade et al., 2024). The relationship between the formulation variables and PS was further illustrated by the three-dimensional response surface plot and contour plot (Figures 3C,D). These plots reveal that PS increased with increasing polymer concentration while decreasing with increasing stirring speed. Smaller microspheres were obtained at lower polymer concentrations combined with higher stirring speeds, which favours improved dispersion and droplet breakup during microparticle formation.

#### 3.1.2 Impact on entrapment efficiency (R2)

EE is a critical quality attribute of microparticulate drug delivery systems, as it reflects the ability of the polymeric matrix to effectively entrap the drug within the particles. Higher EE ensures improved drug loading, minimized drug loss during processing, and better therapeutic performance of the formulation. In the present study, the EE of FCZ-loaded microspheres ranged from 76.23% ± 0.88% to 90.85% ± 0.54% (Table 4), indicating efficient encapsulation of the drug within the polymeric matrix. The relatively higher EE of the formulations demonstrates the reproducibility and reliability of the preparation method employed. The influence of the independent formulation variables on EE was illustrated by the Pareto chart (Figure 4A), which indicates the magnitude and direction of the effects of the selected factors. The Bonferroni limit of 5.06751 was used as a conservative threshold for screening the standardized effects; drug-to-polymer ratio (X_1_) and polymer concentration (X_2_) as they exceeded this threshold, indicating their comparatively stronger influence on EE.

**FIGURE 4:**
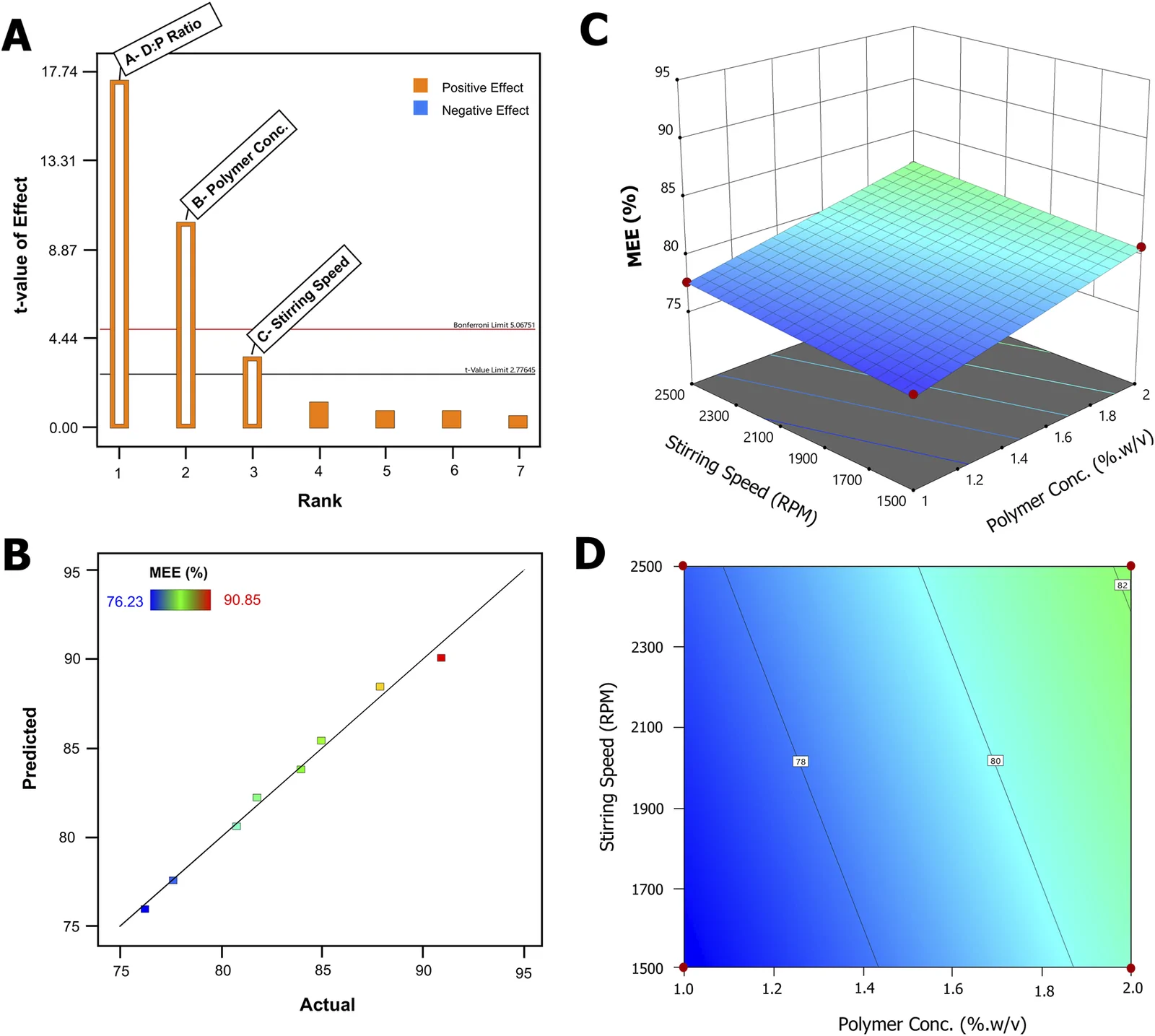
Pareto plot **(A)**, correlation plot comparing actual and predicted values **(B)**, three-dimensional response surface plot **(C)**, and two-dimensional contour plot **(D)** illustrating the effects of stirring speed and polymer concentration while maintaining the drug-to-polymer ratio at the lower level (1:1) on entrapment efficiency of fluconazole-loaded microsphere. The color gradient represents the magnitude of the response (blue: lower and red: higher entrapment efficiency values).

The mathematical model generated for EE is expressed by Equation 20.

$$\text{EE }\left(R2\right)=83.00+3.90.X1+2.31.X2-0.805.X3$$ (20)

The regression model was found to be statistically significant with a model F-value of 146.27 (*p < 0.05*), confirming the adequacy of the model in describing the relationship between the formulation variables and the response. The adjusted *R* ^2^ value of 0.9842 and predicted *R* ^2^ value of 0.9639 indicate excellent agreement between the predicted and experimental values. This shows good agreement between experimental and predicted EE values. The close distribution of data points indicates that the model accurately predicts the response within the studied experimental region (Figure 4B). Furthermore, the adequate precision value of 31.91, which is significantly higher than the desirable value of 4, confirms that the model has an adequate signal and can effectively navigate the design space.

The regression equation indicates that all three formulation variables exerted a positive influence on EE. The drug-to-polymer ratio (X1) exhibited the most significant effect (*p < 0.05*), suggesting that increasing the polymer content relative to the drug enhances the encapsulation efficiency. This phenomenon may be attributed to the effective entrapment of FCZ in the matrix at higher CS levels, which would eventually reduce the surface drug and thereby improve the EE (Avlani et al., 2023). Similarly, polymer concentration (X2) displayed a positive influence on EE, indicating that higher polymer concentrations improve the EE by increasing the viscosity of the polymer solution, which would hamper the drug diffusion into the external phase during microsphere formation. However, although the stirring speed (X3) exhibited a positive effect, its impact was comparatively smaller than that of the other factors. The effect of stirring speed on EE was likely less evident at high polymer proportions and at higher CS concentrations. The interaction between the formulation variables and their effect on EE was further illustrated by the three-dimensional response surface plot and contour plot (Figures 4C,D). These plots demonstrate that EE increases with increasing polymer concentration and drug-to-polymer ratio, while stirring speed has a comparatively smaller influence on the response.

#### 3.1.3 Impact on drug release at the end of 60 min (R3)

Drug release determines the availability of the FCZ at the target site and influences the therapeutic performance of the formulation. In the present study, Q_60_ from the developed FCZ-CMs ranged from 38.63% ± 1.25% to 62.41% ± 1.48% (Figure 5; Table 4). The observed variation among formulations indicates that the selected formulation variables significantly influenced the release behaviour of the drug from the polymeric matrix.

**FIGURE 5:**
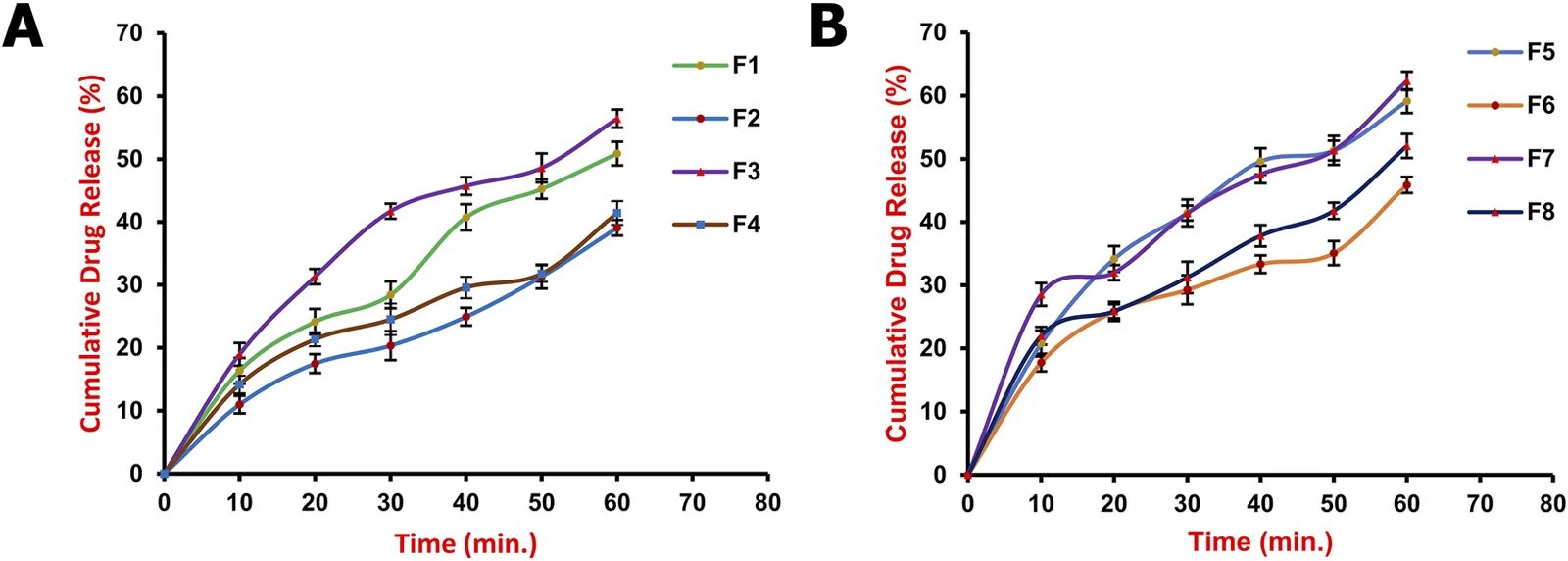
*In vitro* drug release profile of fluconazole-loaded microspheres F1-F4 **(A)** and F5-F8 **(B)**, evaluated using a Type IV flow-through dissolution apparatus in artificial saliva (pH 7.4) under simulated oesophageal conditions (flow rate 1 mL/min, 37 °C ± 0.5 °C); values expressed as mean ± SD (n = 3).

The influence of the formulation variables on Q_60_ was further illustrated using the Pareto chart (Figure 6A), which highlights the magnitude and direction of the effects of the independent variables. The Bonferroni limit of 5.06751 served as the conservative screening threshold, with polymer concentration (X2) and stirring speed (X3) exceeding this threshold and therefore indicating comparatively stronger effects on Q60.

**FIGURE 6:**
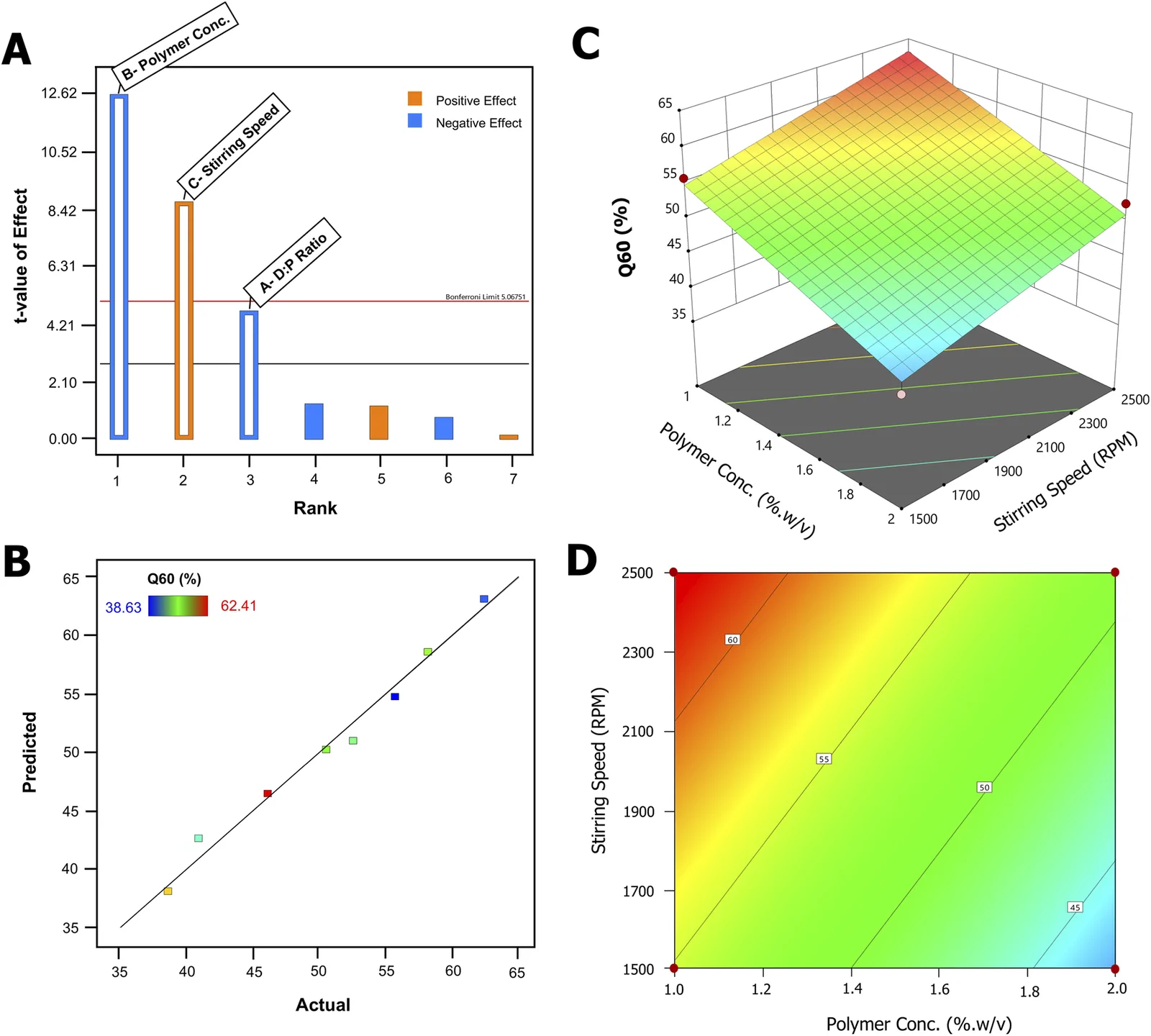
Pareto plot **(A)**, correlation plot comparing actual and predicted values **(B)**, three-dimensional response surface plot **(C)**, and two-dimensional contour plot **(D)** illustrating the effects of stirring speed and polymer concentration while maintaining the drug-to-polymer ratio at the lower level (1:1) on drug release from fluconazole-loaded microspheres. The color gradient represents the magnitude of the response (blue: lower and red: higher drug release values).

The mathematical model generated for Q_60_ (%) by Equation 21.

$$Q_{60}\left(R3\right)=50.64-2.27.X1-6.07.X2+4.18.X3$$ (21)

The regression model demonstrated statistical significance with a model F-value of 85.77 (*p < 0.05*), confirming that the model adequately describes the relationship between the selected variables and the response. The adjusted *R* ^2^ value of 0.9732 and predicted *R* ^2^ value of 0.9388 indicate excellent agreement between the predicted and experimental values. The close distribution of data points accurately predicts the response within the studied experimental region (Figure 6B). Furthermore, the adequate precision value of 26.03, which is well above the desirable value of 4, confirms that the model possesses an adequate signal and can effectively be used to navigate the design space.

The regression equation indicates that polymer concentration (X2) has the most pronounced negative effect on Q_60_, suggesting that an increase in polymer concentration results in a decrease in drug release. This behaviour may be attributed to the formation of a denser polymeric matrix, which is likely to increase the diffusional path length for the drug, thereby retarding its release. Similarly, the drug-to-polymer ratio (X1) displayed a negative influence on drug release, indicating that increasing polymer proportion relative to the drug leads to a slower release rate (Hussain et al., 2026). Conversely, stirring speed (X3) exhibited a positive effect on Q_60_, indicating that higher stirring speeds reduce the PS, thereby promoting the drug release. This effect may be attributed to the formation of smaller microspheres at higher agitation speeds, resulting in an increased surface area that would increase the drug release (Ragheb et al., 2026). The relationship between the independent variables and drug release was further illustrated using three-dimensional response surface plots and contour plots (Figures 6C,D). These plots demonstrate that higher polymer concentrations combined with lower stirring speeds produce formulations with slower drug release, whereas lower polymer concentrations and higher stirring speeds favour faster drug release.

#### 3.1.4 Impact on percentage oesophageal retention of particles (R4)

ORT is an important parameter that determines the performance of FCZ-CMs intended for localized drug delivery in the oesophagus. Higher retention of microspheres on the oesophageal surface ensures prolonged residence time of the formulation at the target site, thereby enhancing local drug availability and therapeutic efficacy. In the present study, ORT_30_ ranged from 32.64% ± 1.25% to 62.16% ± 1.18% (Figure 7; Table 4), indicating that the selected formulation and process variables significantly influenced the retention behaviour of the microspheres.

**FIGURE 7:**
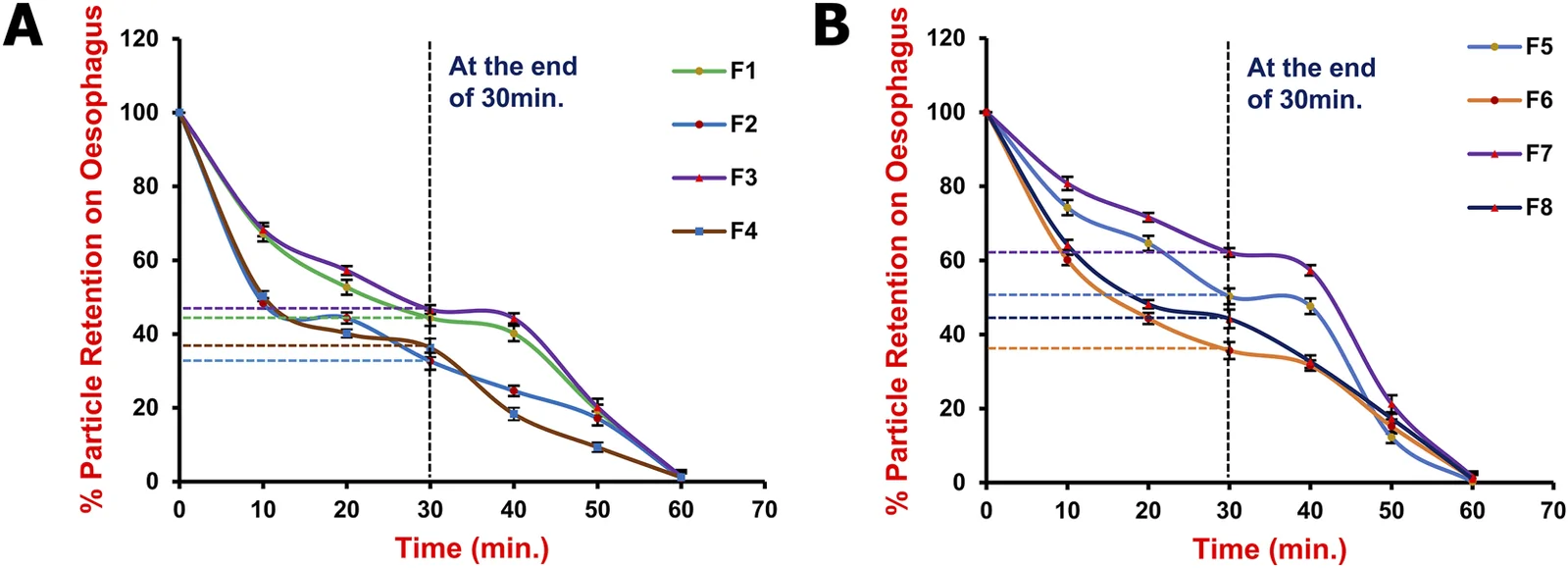
*Ex vivo* particle retention profile of fluconazole-loaded microspheres under simulated physiological oesophageal conditions in a custom-designed flow-through model. Retention behaviour of formulations F1–F4 **(A)** and F5–F8 **(B)** in artificial saliva (pH 7.4) at a flow rate of 1 mL/min and a temperature of 37 °C ± 0.5 °C. Data is expressed as mean ± SD (n = 3).

The relationship between PS, Q_60_, and ORT_30_ of the prepared FCZ-CMs is illustrated in Figure 8. A clear influence of PS on both drug release and mucoadhesive behaviour was observed. Formulations with smaller PS (F5: 34.64 µm and F2: 36.71 µm) exhibited relatively higher Q_60_ (62.41% ± 1.48% and 58.18% ± 2.10%, respectively) along with improved oesophageal retention (62.16% ± 1.18% and 50.32% ± 2.13%). In contrast, larger particles (F4: 89.49 µm and F6: 85.76 µm) demonstrated comparatively lower drug release and reduced retention. A clear inverse relationship was observed, wherein smaller microspheres exhibited higher retention on the mucosal surface with higher drug release, while larger particles displayed comparatively reduced adhesion with relatively reduced drug release. This behaviour can most likely be attributed to the increased specific surface area resulting in enhanced mucoadhesion of smaller particles, which facilitates stronger interactions with the mucin layer, facilitating site-specific drug release to local tissue (Xu et al., 2026). Thus, enhanced mucoadhesion facilitates prolonged residence of the microspheres at the target site, thereby promoting localized drug release and improving therapeutic effectiveness (Tekade et al., 2025). In contrast, larger particles may experience reduced surface interaction owing to larger size or decreased specific surface area, rendering the particles susceptible to detachment under dynamic conditions.

**FIGURE 8:**
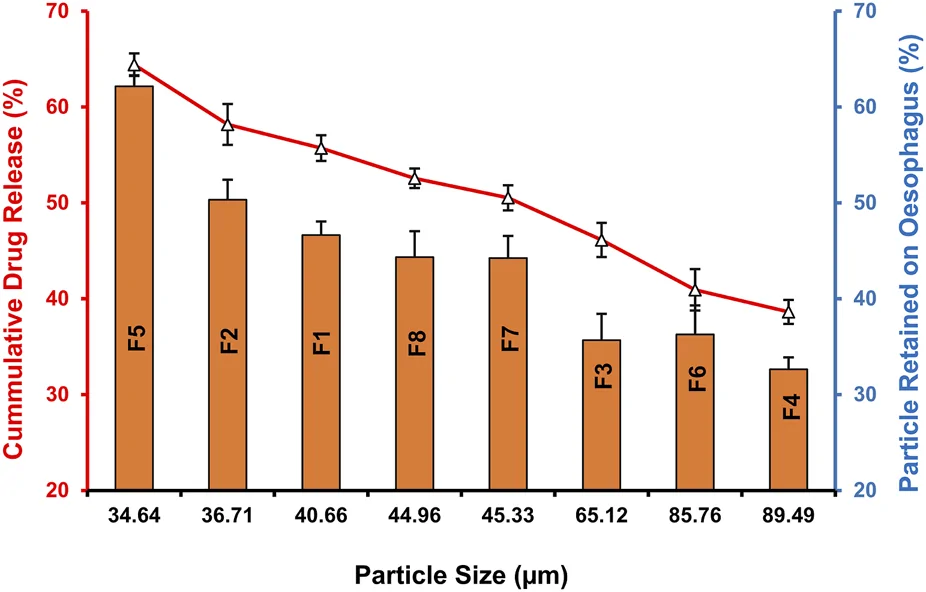
Relationship between particle size, cumulative *in vitro* drug release, and percentage oesophageal retention of fluconazole-loaded chitosan microsphere formulations. The plot illustrates the influence of particle size on both drug release (left y-axis) and oesophageal retention (right y-axis), highlighting that smaller particle sizes are associated with relatively higher retention and enhanced drug release performance. Data are expressed as mean ± SD (n = 3).

Furthermore, PS was significantly influenced by formulation variables, particularly polymer concentration (X2) and stirring speed (X3). An increase in polymer concentration resulted in larger PSs (F4 and F6), which can be attributed to increased viscosity of the polymeric phase, limiting efficient droplet breakup during emulsification. Conversely, higher stirring speeds promoted the formation of smaller and more uniform microspheres (F2 and F5) due to enhanced shear forces. These smaller particles exhibited superior mucoadhesion and drug release performance. FCZ-CMs are likely to be well retained on the mucosal surface by virtue of their cationic nature. CMs are well known for their biocompatibility and biodegradability with cationic charges, making them an attractive platform for site-specific antimicrobial applications. A synergistic antimicrobial effect of CS has been observed with FCZ in previously reported literature (Lu et al., 2026). Overall, the findings demonstrate a significant interdependence between PS, drug release, and mucoadhesive behaviour. The perfect balance of these parameters is essential for achieving prolonged oesophageal residence and effective localized drug delivery. The results confirm that the developed FCZ-CMs possess adequate mucoadhesive potential to facilitate localized drug release, maintaining sufficient retention at the target site.

The effect of formulation variables on oesophageal retention was further elucidated using Pareto analysis (Figure 9A), which quantitatively highlights the contribution of each factor. The Bonferroni limit of 5.06751 served as the conservative screening threshold, with polymer concentration (X2) exceeding this limit and thereby exhibiting the strongest negative effect on ORT_30_, indicating that higher polymer levels adversely affect retention. This behaviour can be attributed to the formation of denser and larger microspheres with reduced surface accessibility of functional groups, thereby limiting effective interfacial interaction with the mucosal layer. In contrast, stirring speed (X3) exhibited a positive effect, suggesting that increased stirring speed facilitates smaller particles, which enhances retention performance. This improvement is associated with enhanced shear forces during emulsification, leading to the formation of finer and more uniformly distributed microspheres with improved surface characteristics conducive to mucoadhesion (Zhao et al., 2026). The drug-to-polymer ratio (X1) showed a comparatively lesser negative effect, indicating that higher drug loading slightly compromises retention, possibly due to reduced availability of polymer chains necessary for effective adhesion. Collectively, these findings emphasize that formulation variables govern oesophageal retention primarily through their influence on particle characteristics and surface interactions, with polymer concentration emerging as the most critical factor.

**FIGURE 9:**
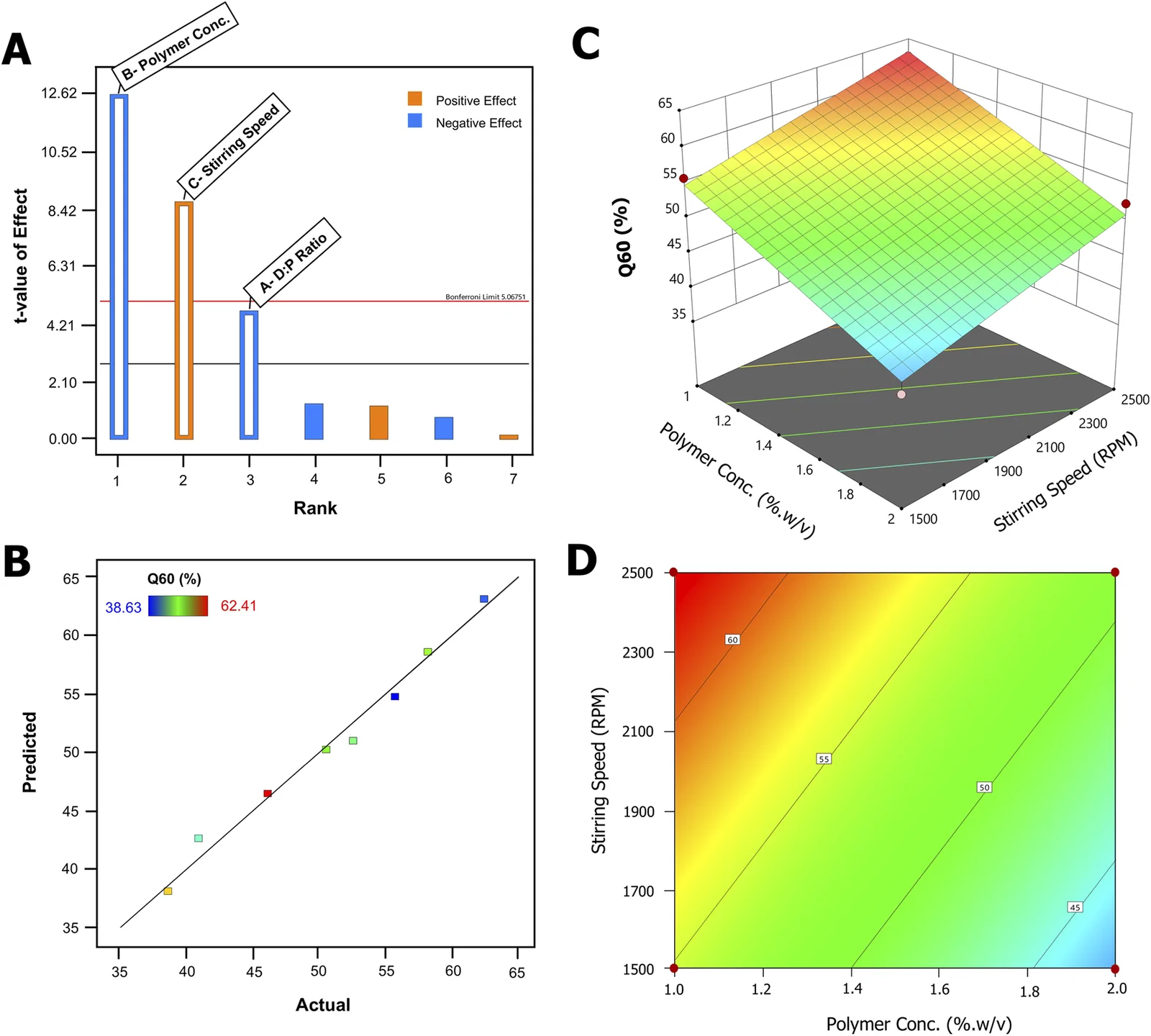
Pareto plot **(A)**, correlation plot comparing actual and predicted values **(B)**, three-dimensional response surface plot **(C)**, and two-dimensional contour plot **(D)** illustrating the effects of stirring speed and polymer concentration while maintaining the drug-to-polymer ratio at the lower level (1:1) on percentage retention of microsphere on the esophagus. The color gradient represents the magnitude of the response (blue: lower and red: higher adherence values).

The mathematical relationship describing the effect of formulation variables on ORT_30_ (%) in terms of coded factors is expressed by Equation 22.

$${\text{ORT}}_{30}\left(R4\right)=44.04-3.29.X1-6.83.X2+4.06.X3$$ (22)

The regression model was found to be statistically significant with a model F-value of 18.32 (*p < 0.05*), confirming that the model adequately describes the relationship between formulation variables and the response. The adjusted *R* ^2^ value of 0.8813 and predicted *R* ^2^ value of 0.7287 indicate good agreement between predicted and experimental values (Figure 9B). Furthermore, the adequate precision value of 12.23, which is higher than the desirable value of 4, confirms that the model possesses an adequate signal and can be effectively used to navigate the design space. The regression equation indicates that polymer concentration (X2) has the most pronounced negative effect on oesophageal retention. Decreasing the polymer concentration would result in reduced PS of the CMs that would promote better retention at the surface of the mucosa. Likewise, increasing the stirring speed would decrease the PS and, therefore, the retention of the CMs. Similarly, the drug-to-polymer ratio (X1) that had the least negative influence, indicating that lower polymer content relative to drug may increase the adhesive characteristics of the microspheres. Conversely, stirring speed (X3) exhibited a positive effect on ORT_30_, suggesting that higher stirring speeds enhance oesophageal retention.

This phenomenon may be attributed to the formation of smaller and more uniformly distributed microspheres at higher agitation speeds, which increases the effective surface area available for interaction with the mucosal layer (Bhalala et al., 2026). The relationship between formulation variables and oesophageal retention was further illustrated by the three-dimensional response surface and contour plots (Figures 9C,D). These plots indicate that higher stirring speeds combined with moderate polymer concentrations favour improved oesophageal retention, whereas higher polymer concentrations and lower stirring speeds result in reduced particle adherence.

The *ex vivo* retention model employed in the present study provides preliminary evidence of the retention potential of FCZ-CMs on goat oesophageal mucosa. The use of freshly excised goat oesophageal mucosa provides a native biological surface containing the structural and mucosal features necessary to assess microsphere–mucosa interactions, while the modified USP disintegration apparatus introduces a controlled cyclic mechanical challenge under simulated salivary conditions. Thus, the model provides a useful preliminary assessment of the relative retention potential of the developed CMs before progression for further *in vivo* evaluation. However, the model does not fully simulate the complex physiological environment of the human oesophagus. Particularly, physiological peristaltic contractions, continuous and variable salivary flow, mucus turnover, swallowing dynamics, mucosal clearance, and inter-individual differences in oesophageal anatomy and mucus composition cannot be completely reproduced in the present relatively static *ex vivo* setup. Therefore, the observed ORT_30_ values should be interpreted as indicators of relative mucoadhesive retention under controlled *ex vivo* conditions rather than direct predictors of human oesophageal residence time. Nevertheless, the observed retention rate offers a rough estimate of the potential of the FCZ-CMs to remain associated with oesophageal mucosa under a dynamic mechanical challenge that warrants for further *in vivo* investigation.

### 3.2 Optimization of formulation

A desirability-based numerical optimization successfully identified optimal FCZ-CMs as mentioned in Table 5. The desirability plot and the associated overlay plot are presented in Figures 10A,B, respectively. A desirability value closer to 1.0 indicates greater reliability of the predicted response.

**TABLE 5:** Regression analysis and model fit statistics for particle size, entrapment efficiency, *in vitro* drug release, and percentage oesophageal retention of particles.

Responses	R^2^	Adj. R^2^	Pred. R^2^	SD	Adeq. Precision	%CV	Remarks
R1: PS^a^ (µm)	0.8525	0.7795	0.5968	10.33	8.21	18.66	Significant
R2: EE^b^ (%)	0.9910	0.9842	0.9639	0.62	31.92	0.75	Significant
R3: Q_60_^c^ (%)	0.9847	0.9732	0.9388	1.36	26.04	2.69	Significant
R4: ORT_30_^d^ (%)	0.9322	0.8813	0.7287	3.28	12.23	7.49	Significant

^a^ Particle size.

^b^ Entrapment efficiency.

^c^ In vitro percentage drug release at the end of 60 min.

^d^ In vitro percentage oesophageal retention of particles at the end of 30 min.

**FIGURE 10:**
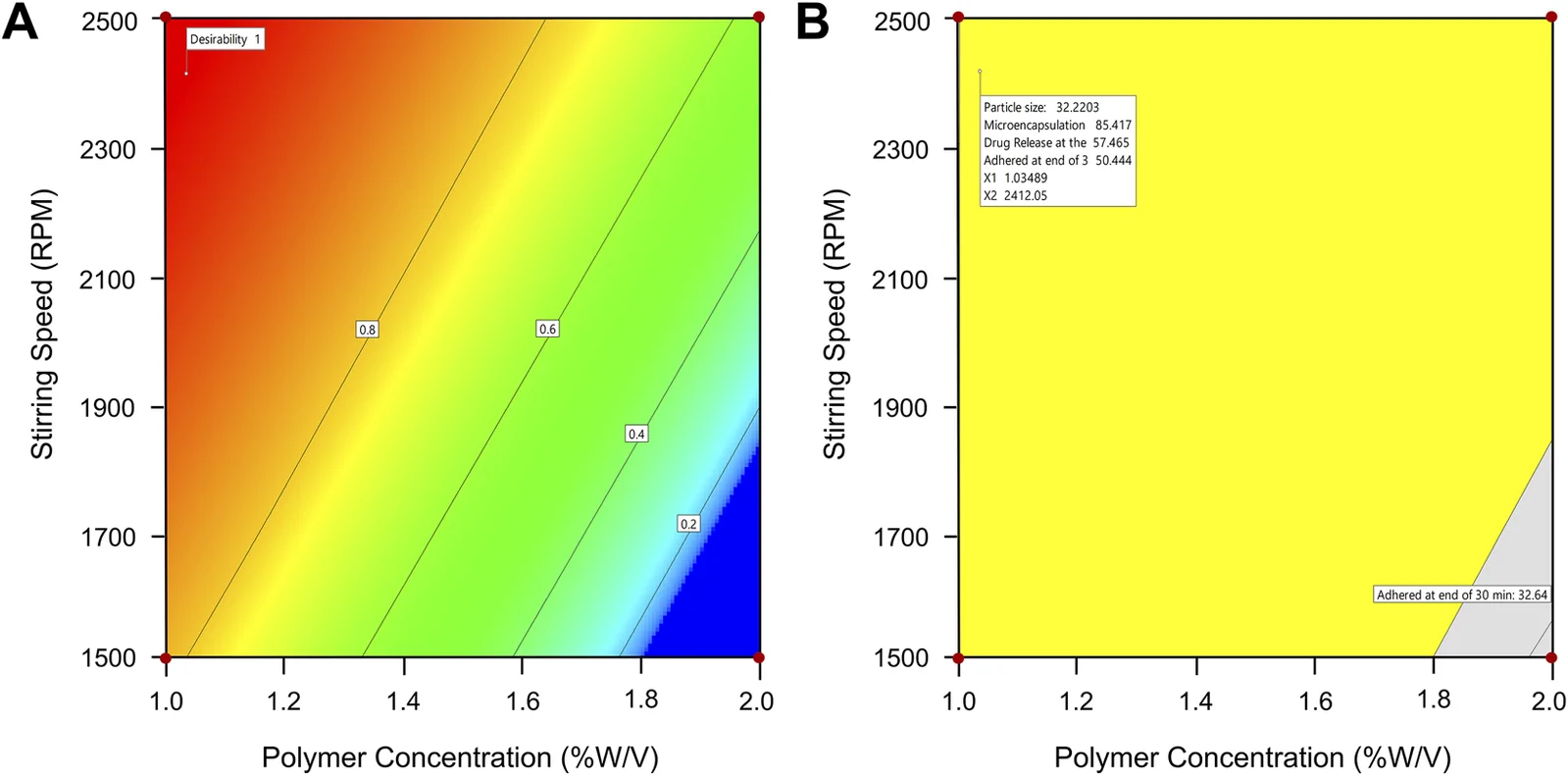
Optimization plots for formulation design **(A)** desirability response surface plot showing the distribution of desirability values (blue indicating low desirability and red indicating high desirability) obtained through numerical optimization **(B)** overlay design space plot identifying the optimal formulation region (yellow area) and non-optimal region (grey area) based on variations in stirring speed and polymer concentration while maintaining the drug-to-polymer ratio at a lower level (1:2).

To validate the reliability of the numerical optimization results, an FCZ-CMs (F9) was prepared using the predicted optimal conditions, namely, a drug-to-polymer ratio of 1:2, polymer concentration of 1.035%, and stirring speed of 2,412 rpm. The experimentally obtained response parameters were then compared with the predicted values generated by the design model (Table 6). The optimised formulation exhibited a PS (31.81 ± 1.14 µm), EE (84.12% ± 2.15%), Q_60_ (58.46% ± 1.89%), and ORT_30_ (51.22% ± 2.11%). F9 exhibited low prediction errors for PS (1.27%), EE (1.52%), Q_60_ (1.72%), and ORT_30_ (1.55%), confirming the accuracy of the optimization model. The prediction errors were low (*<2%*), confirming the validity of polynomial regression models.

**TABLE 6:** Composition of the optimised formulation and comparison of experimentally obtained response parameters and product quality attributes with the corresponding predicted values.

Run	Optimal settings X_1_:X_2_:X_3_	Responses	Experimental value	Predicted value	Prediction error (%)
F9**	X1: 1:2 ratio X2: 1.035% X3: 2,412 rpm	R1 (µm)	31.81 ± 1.14	32.22	1.27
		R2 (%)	84.12 ± 2.15	85.42	1.52
		R3 (%)	58.46 ± 1.89	57.47	1.72
		R4 (%)	51.22 ± 2.11	50.44	1.55

** Optimised batch (F9) produced using the casting solution of the above composition.

### 3.3 Characterization of optimised fluconazole-loaded chitosan microspheres

Following optimization, the formulation was further subjected to detailed physicochemical and structural characterization, including FTIR, DSC, XRD, and SEM analyses, to investigate potential drug–polymer interactions, crystallinity changes, and surface morphology of the microspheres. In addition, release kinetic modelling was evaluated to better understand the release mechanism and to characterize the release mechanism of the optimised FCZ-CMs (F9). These investigations collectively provide comprehensive insight into the performance and structural characteristics of the optimised microsphere system.

#### 3.3.1 Fourier transform infrared spectroscopy

Compatibility between FCZ and the selected polymer (i.e., CS) was evaluated using FTIR to identify any potential chemical interactions that could affect formulation stability (Table 7). The FTIR spectrum of FCZ exhibited characteristic absorption bands corresponding to O–H stretching (3,469.3 cm^-1^), aromatic C–H stretching (3,017.0 cm^-1^), CH_2_ stretching (2,965.9 cm^-1^), C=C stretching (1,616.0 cm^-1^), C–O–C stretching (1,128.1 cm^-1^), and C–F stretching (1,217.8 cm^-1^). The observed results align closely with the characteristic peaks of FCZ previously reported in the literature (Helal et al., 2022). CS displayed characteristic peaks at 3,586.9 cm^-1^ (O–H stretching) and 3,238.8 cm^-1^ (N–H stretching), values are in good agreement with reported values for CS (Patil et al., 2026). The physical mixture and optimised FCZ-CMs (F9) retained the principal peaks of both FCZ and CS with only minor shifts, indicating the absence of significant chemical interaction and confirming the compatibility of the drug with the polymeric matrix.

**TABLE 7:** Fourier transform infrared characteristics and vibrational bands of fluconazole, chitosan, physical mixture, and optimised microparticle (F9).

Characteristic peak position (cm^-1^)	Functional group	Fluconazole (cm^-1^)	Chitosan (cm^-1^)	Physical mixture (cm^-1^)	Optimised microsphere F9 (cm^-1^)
3,600–3,400	O–H stretching	3,469.3	3,586.9	3,468.1	3,586.9
3,300–3,200	N–H stretching	–	3,238.8	3,234.2	3,238.2
3,050–3,000	Arom. C-H stretch	3,017.1	–	3,019.9	3,019.9
3,000–2,850	CH_2_ stretching	2,965.9	2,872.4	2,961.1	2,849.3
1,650–1,600	C=C stretching	1,128.1	–	1,129.1	1,128.9
1,250–1,200	C–F stretching	1,616.2	–	1,614.1	1,614.4
1,150–1,100	C–O–C stretching	1,217.8	–	1,219.7	1,218.4

#### 3.3.2 Morphological evaluation of fluconazole microspheres

SEM was performed to evaluate the surface morphology and structural characteristics of the optimised FCZ-CMs (F9). The SEM micrographs revealed that the prepared microspheres were predominantly spherical in shape and uniformly distributed, indicating successful formation of the particulate system. At lower magnification (Figure 11A), the microspheres appeared discrete and well separated, suggesting minimal aggregation during the preparation process. Higher magnification images (Figure 11B) further demonstrated that the microspheres possessed a well-defined rough and wrinkled surface morphology, which could be attributed to solvent evaporation and polymer precipitation during the microsphere formation process. Rough-surfaced CS particles produced by ionotropic gelation techniques have been reported to exhibit improved mucoadhesive properties (Nikou et al., 2026; Amin and Boateng, 2022). The presence of such surface irregularities may contribute to increased interfacial contact with the mucosal surface and may therefore support the observed mucoadhesive retention. Overall, the SEM analysis confirms the successful formation of FCZ-CMs with desirable morphological characteristics suitable for oesophageal targeting.

**FIGURE 11:**
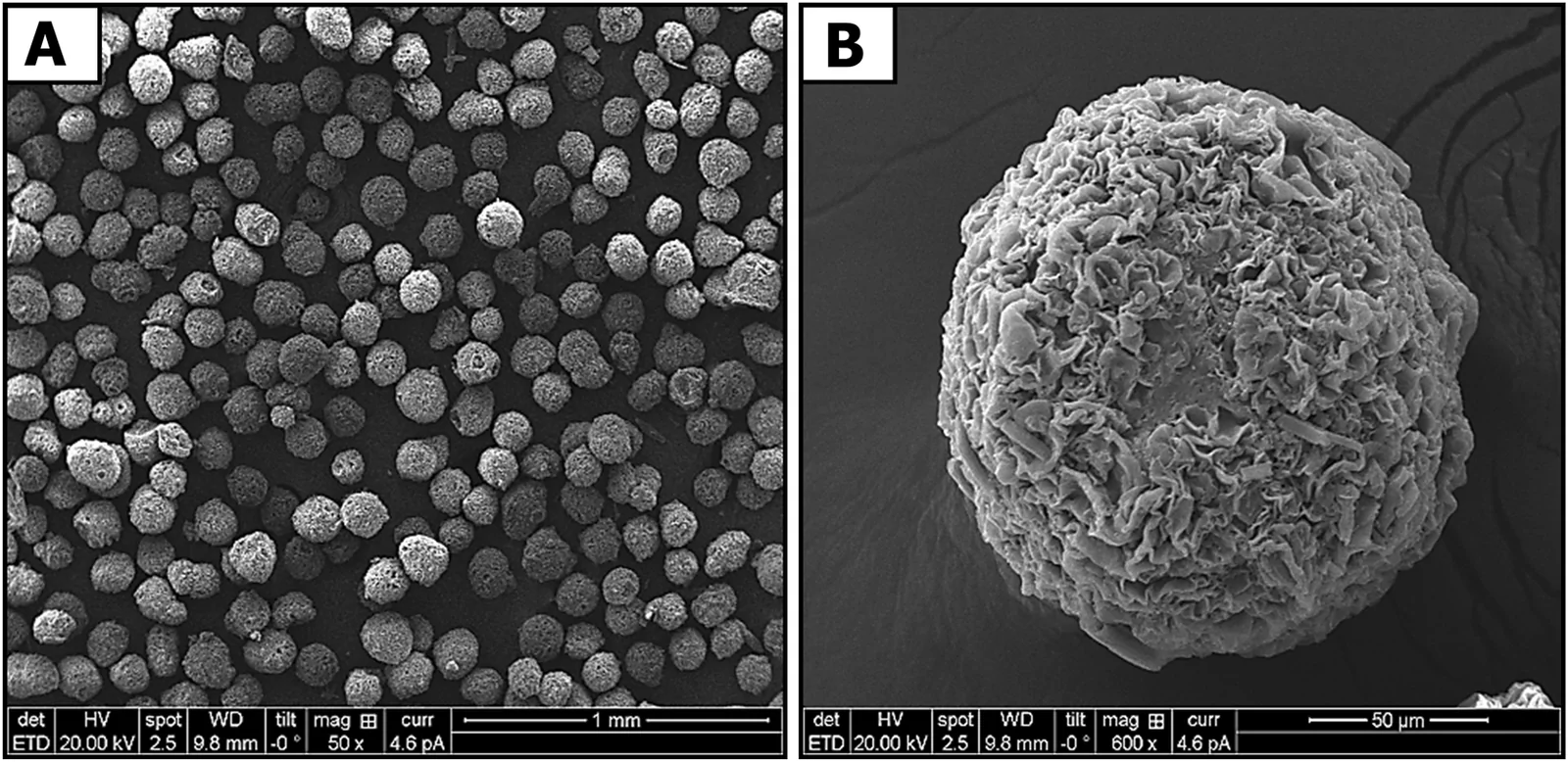
Scanning electron microscopic analysis of fluconazole-loaded at 1 mm **(A)** and 50 µm **(B)** magnification, respectively, of optimised microsphere formulation (F9).

#### 3.3.3 Thermal analysis by differential scanning calorimetry (DSC)

DSC was employed to investigate the thermal behaviour and possible interactions between FCZ and excipients in the prepared microspheres. DSC is a widely used thermal analysis technique that measures heat flow associated with phase transitions such as melting, crystallization, and glass transition of materials. The thermogram of pure FCZ (Figure 12A) exhibited a sharp endothermic peak at 141.37 ° C, corresponding to its melting point, which confirms the crystalline nature of the drug and is consistent with reported literature values (Vithlapara et al., 2024). The DSC thermogram of CS showed a broad endothermic peak around 92.31 ° C, which can be attributed to the evaporation of bound moisture present in the polymer matrix. In the thermogram of the physical mixture (PM), the characteristic melting peak of FCZ was still observed at 143.51 ° C, along with a broad peak near 90.93 ° C, indicating that the drug remained stable and that no significant chemical interaction occurred between FCZ and the CS. However, in the optimised FCZ-CMs (F9), the sharp melting peak of FCZ was significantly reduced, indicating a predominantly amorphous or molecularly dispersed state of the FCZ within the polymeric matrix. This change in thermal behaviour indicates successful incorporation of FCZ into the CS microspheres without any chemical incompatibility, supporting the stability of the developed formulation.

**FIGURE 12:**
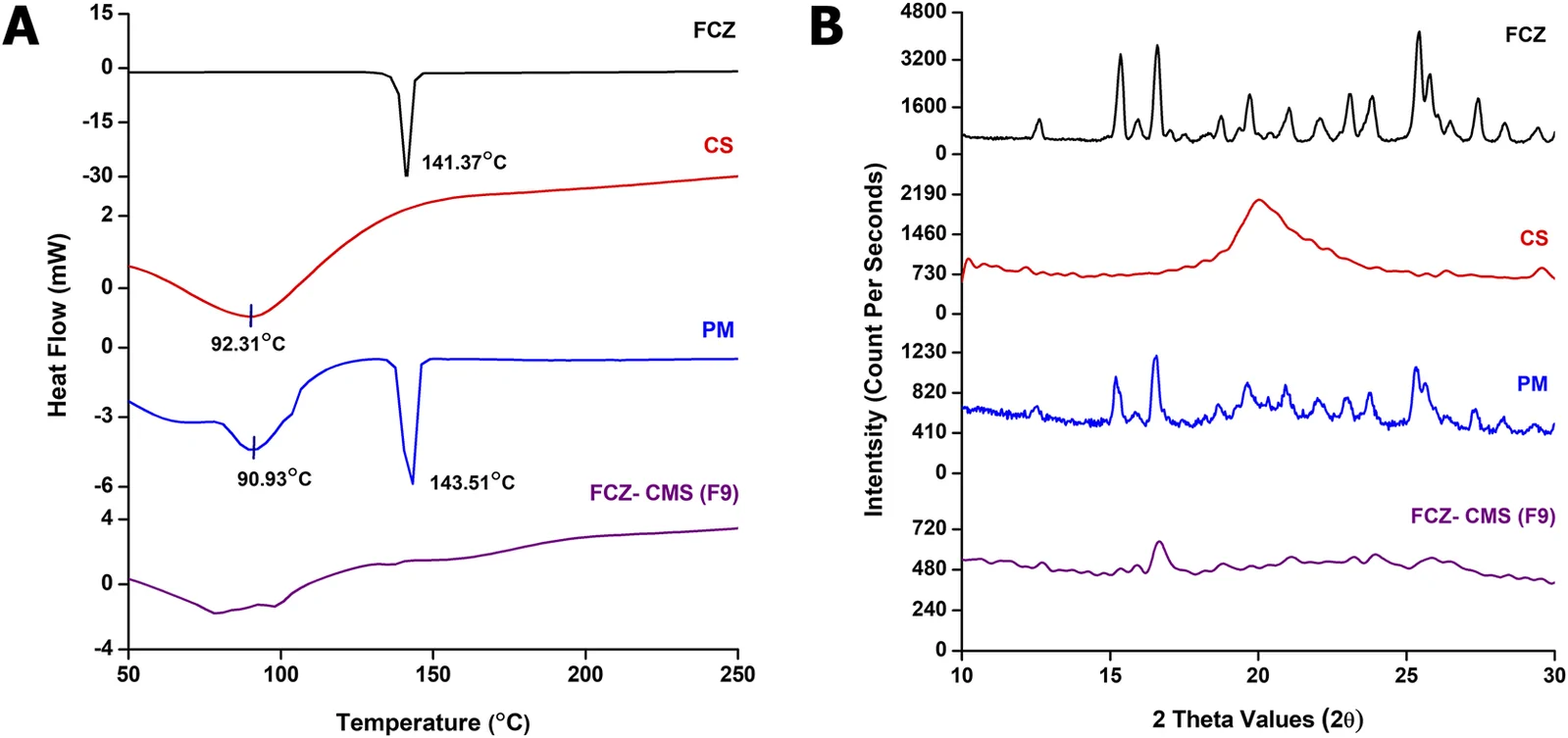
Thermal characterization by Differential Scanning Calorimetry **(A)** and crystalline characterization by X-Ray Diffraction **(B)**, of Fluconazole (FCZ), Chitosan (CS), Physical Mixture of FCZ and Excipients (PM), optimised FCZ-loaded microspheres (FCZ-CMs F9).

#### 3.3.4 Crystallinity assessment by X-Ray diffractometry (XRD)

XRD analysis was performed to evaluate the crystalline characteristics of FCZ, CS, physical mixture (PM), and the optimised FCZ-CMs (F9) as depicted in Figure 12B. FCZ displayed numerous sharp diffraction peaks clustered between 15° and 27° 2θ, consistent with those reported in the literature (Vithlapara et al., 2024). A prominent peak was observed at 25.4° (2θ) with a maximum intensity of 4,150.30 cps, which was considered 100% relative crystallinity. In the physical mixture, the intensity of the same characteristic peak was significantly reduced to 1,079.98 cps, corresponding to a relative crystallinity of 26.02%, indicating partial disruption of the crystalline lattice due to the presence of the polymer matrix. Furthermore, the optimised FCZ-CMs (F9) showed a substantial reduction in peak intensity to 549.42 cps, corresponding to only 13.24% crystallinity, demonstrating a pronounced decrease in the crystalline nature of FCZ. The marked reduction in peak intensity and disappearance of several characteristic diffraction peaks suggest that the drug was successfully dispersed within the CS polymeric matrix in a predominantly amorphous or molecularly dispersed state. Such a transformation from crystalline to amorphous form is advantageous as it can improve drug solubility, dissolution behaviour, and overall formulation performance.

#### 3.3.5 Kinetic modelling and mechanistic interpretation of *in vitro* drug release profiles

The *in vitro* drug release kinetics of the FCZ-CMs (F1–F8) and the optimised batch (F9) were evaluated using first-order, Higuchi, and Korsmeyer–Peppas models to elucidate the underlying release mechanism (Table 8). The first-order model showed high correlation coefficients, indicating that drug release from the microspheres was partially dependent on FCZ concentration within the polymeric matrix. The Higuchi model exhibited strong linearity for most formulations, suggesting that drug release was predominantly governed by a diffusion-controlled mechanism from the polymer matrix.

**TABLE 8:** Curve fitting results of *in vitro* dissolution data for the fluconazole-loaded microsphere formulation using various kinetic models.

Batch	First order plot		Higuchi plot		Korsmeyer-peppas plot		
	K_1_ ^a^ (min^-1^)	*R* ^*2*^	K_H_ ^b^ (%.min^-1/2^)	*R* ^*2*^	‘n’^c^ value	K_P_ ^d^ (min^-n^)	*R* ^*2*^
F1	0.20 ± 0.003	0.986 ± 0.012	6.341 ± 0.48	0.972 ± 0.012	0.647 ± 0.03	0.36 ± 0.01	0.979 ± 0.013
F2	0.21 ± 0.003	0.977 ± 0.044	7.867 ± 0.82	0.950 ± 0.025	0.670 ± 0.02	0.25 ± 0.01	0.975 ± 0.024
F3	0.19 ± 0.005	0.965 ± 0.013	5.624 ± 0.57	0.990 ± 0.089	0.590 ± 0.01	0.50 ± 0.02	0.979 ± 0.013
F4	0.21 ± 0.007	0.956 ± 0.067	7.561 ± 0.12	0.976 ± 0.061	0.551 ± 0.06	0.43 ± 0.01	0.974 ± 0.012
F5	0.19 ± 0.002	0.969 ± 0.057	5.275 ± 0.56	0.993 ± 0.042	0.567 ± 0.01	0.58 ± 0.02	0.985 ± 0.034
F6	0.20 ± 0.004	0.883 ± 0.010	6.986 ± 0.78	0.984 ± 0.047	0.474 ± 0.06	0.65 ± 0.03	0.943 ± 0.028
F7	0.19 ± 0.002	0.931 ± 0.026	4.859 ± 0.96	0.977 ± 0.034	0.432 ± 0.07	1.06 ± 0.02	0.961 ± 0.024
F8	0.19 ± 0.001	0.954 ± 0.037	6.189 ± 0.11	0.977 ± 0.024	0.465 ± 0.02	0.78 ± 0.01	0.937 ± 0.042
F9**	0.19 ± 0.006	0.950 ± 0.033	5.363 ± 0.43	0.990 ± 0.066	0.414 ± 0.04	1.07 ± 0.04	0.969 ± 0.021

^**^ Optimised fluconazole-loaded chitosan microsphere (F9).

^a^ First-order release rate constant (×10–1. min-1).

^b^ Higuchi release rate constant (%.min-1/2), and.

^c^ Release exponent and.

^d^ Korsmeyer-Peppas release rate constant (×10–1. min^-n^). All data in the table represent the mean ± SD (n = 3).

Further analysis using the Korsmeyer–Peppas exponent suggested a predominantly diffusion-controlled to anomalous transport mechanism, depending on formulation, indicating that both drug diffusion and polymer relaxation may contribute to release. Among the studied batches, the optimised formulation F9 showed good model fitting with *R* ^2^ values of 0.950 (first-order), 0.990 (Higuchi), and 0.969 (Korsmeyer–Peppas) with a release exponent n = 0.414, suggesting a diffusion-dominated release mechanism with contribution from polymer matrix relaxation. These results indicate that the developed microsphere system can provide site-specific drug release, which is advantageous for improving the therapeutic performance of fluconazole in oesophageal targeting applications.

### 3.4 Characterization of optimised FCZ-CMs-loaded orodispersible tablet

#### 3.4.1 Pre-compression characterisation of the powder

The micromeritic properties of the powder blends prepared for the FCZ-CMs-loaded orodispersible tablets (ODTs) are presented in Table 9. The powder blend exhibited lower values of the angle of repose, Carr’s compressibility index, and Hausner ratio, indicating improved flowability and compressibility. The enhanced micromeritic behaviour can be attributed to the PS and more spherical morphology of the CMs, which reduced interparticle friction and improved particle flow and packing. Similar improvements in powder flow and compression characteristics following microencapsulation have been reported previously for CS-based multiparticulate systems and other directly compressible formulations (Agnihotri, Mallikarjuna, and Aminabhavi, 2004).

**TABLE 9:** Micromeritic properties of the powder blend before tablet compression.

Micromeritic parameter	Observations
Bulk density (g/cm^3^)	0.48 ± 0.01
Tapped density (g/cm^3^)	0.55 ± 0.02
Carr’s compressibility index (%)	12.73 ± 0.54
Hausner ratio	1.15 ± 0.02
Angle of repose	23.84 ± 0.36

Data are expressed as mean ± standard deviation (SD) (n = 3).

The observed Carr’s compressibility index and Hausner ratio further confirmed the good flow characteristics of the optimised powder blend. These parameters are widely accepted indicators of powder flowability and packing behaviour, and lower values are associated with improved die filling and compression performance (Tharanon et al., 2024). Collectively, the results demonstrate that incorporation of FCZ into CMs significantly improved the micromeritic properties of the formulation, making the optimised powder blend suitable for direct compression into ODTs.

#### 3.4.2 Post-compression evaluation

The post-compression properties of the FCZ-CMs-loaded ODTs are presented in Table 10. The formulation exhibited satisfactory physical quality and complied with the pharmacopeial acceptance criteria for all evaluated parameters.

**TABLE 10:** Post-compression characteristics of the prepared orodispersible tablets.

Parameter	Observations
Weight variation (mg)	160.2 ± 0.3
Hardness (kg/cm^2^)	3.42 ± 0.15
Thickness (mm)	3.24 ± 0.04
Friability (%)	0.36 ± 0.03
Wetting time (s)	19.62 ± 0.51
Disintegration time (s)	23.83 ± 0.86
Drug content (%)	99.14 ± 0.58

Data are expressed as mean ± standard deviation (SD) (n = 3).

The tablets were found to display uniform weight (160.2 ± 0.3 mg), by virtue of good flow of the powder blend leading to uniform die filling during compression. The ODTs possessed adequate hardness (3.42 ± 0.15 kg/cm^2^) and uniform thickness (3.24 ± 0.04 mm), providing sufficient mechanical strength to withstand handling without compromising rapid disintegration (Taylor and Aulton, 2022). The friability was 0.36% ± 0.03%, well below the pharmacopeial limit of 1%, confirming excellent mechanical integrity (Shah, Madan, and Agrawal, 2012). The ODTs exhibited a DC of 99.14% ± 0.58%, indicating uniform distribution of FCZ-CMs within the tablet matrix and high content uniformity. Collectively, these findings demonstrate that the developed ODTs possessed the requisite mechanical strength, rapid hydration and disintegration characteristics, and satisfactory content uniformity, making them a suitable carrier for localized oesophageal delivery of fluconazole. Microcrystalline cellulose and mannitol improved powder compressibility and tablet integrity, while the water-soluble nature of mannitol has been reported to enhance the organoleptic properties of ODTs by imparting a pleasant cooling sensation in the oral cavity (Lin et al., 2025; Berkenkemper et al., 2020).

#### 3.4.3 Wetting time

The ODTs exhibited a short wetting time of 19.62 ± 0.51s as depicted in Figure 13. Wetting time is an important factor influencing tablet disintegration and subsequent FCZ dissolution. The ODT exhibited rapid wetting, indicating efficient penetration of the aqueous medium into the tablet matrix. Rapid wetting is a critical quality attribute of ODTs because it facilitates immediate hydration of the tablet surface and serves as the initial step governing subsequent tablet disintegration (Berkenkemper et al., 2020). The wetting behaviour, particularly in the presence of crospovidone and croscarmellose sodium, promotes rapid water uptake through swelling and capillary (wicking) mechanisms, while the high aqueous solubility of mannitol facilitates efficient penetration of the aqueous medium throughout the tablet matrix (Rojas, Guisao, and Ruge, 2012; Wang et al., 2025). Similar short wetting has been reported for directly compressed ODTs containing hydrophilic excipients and multifunctional superdisintegrants (Pabari and Ramtoola, 2012).

**FIGURE 13:**
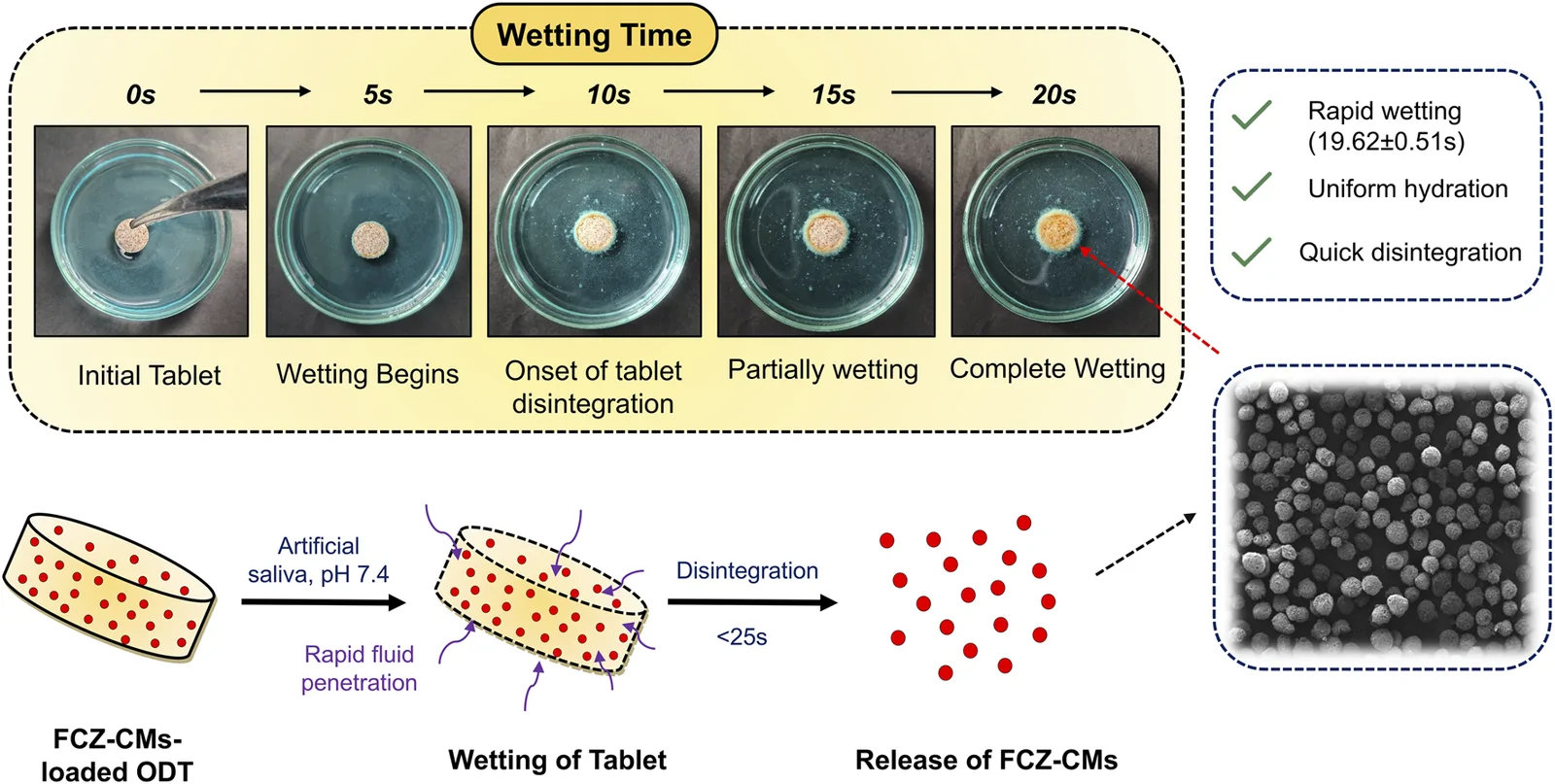
Wetting behaviour and proposed mechanism of the fluconazole-loaded chitosan microsphere (FCZ-CMs) orodispersible tablet (ODT). Progressive hydration in simulated salivary fluid resulted in complete wetting within 19.62 ± 0.51s, followed by rapid tablet disintegration (<25s) and release of FCZ-CMs, facilitating their subsequent oesophageal mucosal deposition.

#### 3.4.4 *In vitro* disintegration time

The ODTs exhibited an *in vitro* DT of 23.83 ± 0.86 s. The short DT closely correlated with the wetting behaviour of both formulations, indicating that rapid penetration of the aqueous medium into the tablet matrix facilitated efficient tablet disintegration. Wetting is considered the initial step governing tablet disintegration (Pabari and Ramtoola, 2012). The rapid disintegration observed for ODTs can be attributed to the synergistic action of crospovidone and croscarmellose sodium, which promoted rapid tablet hydration through swelling and capillary (wicking) mechanisms, thereby facilitating efficient tablet dispersion. Similar findings have been reported for directly compressed ODTs formulated with multifunctional super-disintegrants, where enhanced wettability and rapid liquid penetration resulted in efficient tablet disintegration (Markl and Zeitler, 2017). Furthermore, the DT of the optimised ODT was below the pharmacopoeial limit of 30s, confirming its suitability as an orodispersible dosage form.

#### 3.4.5 Drug content

The DC of the prepared ODTs was found to be 99.14% ± 0.58%. The high FCZ content indicated good FCZ incorporation and low FCZ loss during blending and compression (Taylor and Aulton, 2022). The high DC achieved with the optimised formulation further demonstrates the reproducibility of the direct compression process due to efficient incorporation of FCZ-CMs into the tablet matrix. Similar findings have been reported for directly compressed microsphere-based tablet formulations, where optimised blending and processing conditions ensured uniform drug distribution and dosage accuracy (Berkenkemper et al., 2020).

#### 3.4.6 *In vitro* dissolution study

The *in vitro* dissolution profiles of the optimised FCZ-CMs (F9) and the corresponding FCZ-CMs-loaded ODTs were compared to evaluate the influence of tablet compression on drug release. The CMs exhibited a cumulative drug release of 58.46% ± 1.89% at 60min, while the corresponding tablet ODTs released 53.16% ± 1.24% over the same period. Although ODTs exhibited a marginally lower cumulative drug release than F9, the difference was not statistically significant (*p > 0.05*), indicating that direct compression did not affect the release behaviour of FCZ. To further compare the dissolution profiles, the difference factor (*f*_*1*_) and similarity factor (*f*_*2*_) were calculated. The calculated values (*f*_*1*_ = 8 and *f*_*2*_ = 80) fall within the accepted criteria (*f*_*1*_ ≤ 15 and *f*_*2*_ ≥ 50), confirming a high degree of similarity between the dissolution profiles of F9 and ODTs. These findings demonstrate that the direct compression process of the optimised FCZ-CMs into an ODT did not significantly modify the release characteristics of the entrapped drug. The slight reduction in cumulative drug release observed for ODTs may be attributed to the transient diffusion barrier imposed by the tablet matrix before complete wetting and disintegration. However, owing to the rapid wetting (19.62 ± 0.51s) and fast disintegration (23.83 ± 0.86s) of the tablet, this effect was negligible and did not alter the intrinsic release behaviour of the microspheres. Overall, these results confirm that the controlled-release characteristics of FCZ from the optimised CS microspheres were successfully preserved following their conversion into ODTs, validating the robustness of the formulation strategy for localized oesophageal drug delivery.

Based on the combined findings, a proposed delivery pathway for the FCZ-CMs-loaded ODT is illustrated in Figure 14. The schematic depicts rapid wetting and disintegration of the ODT, release of the optimised FCZ-CMs, subsequent deposition and mucoadhesive retention of the microspheres at the oesophageal mucosa, and controlled local release of FCZ. The proposed oesophageal retention component is based on the *ex vivo* evaluation of the optimised FCZ-CMs, whereas the rapid disintegration of ODTs results in liberation of microspheres to facilitate oesophageal deposition.

**FIGURE 14:**
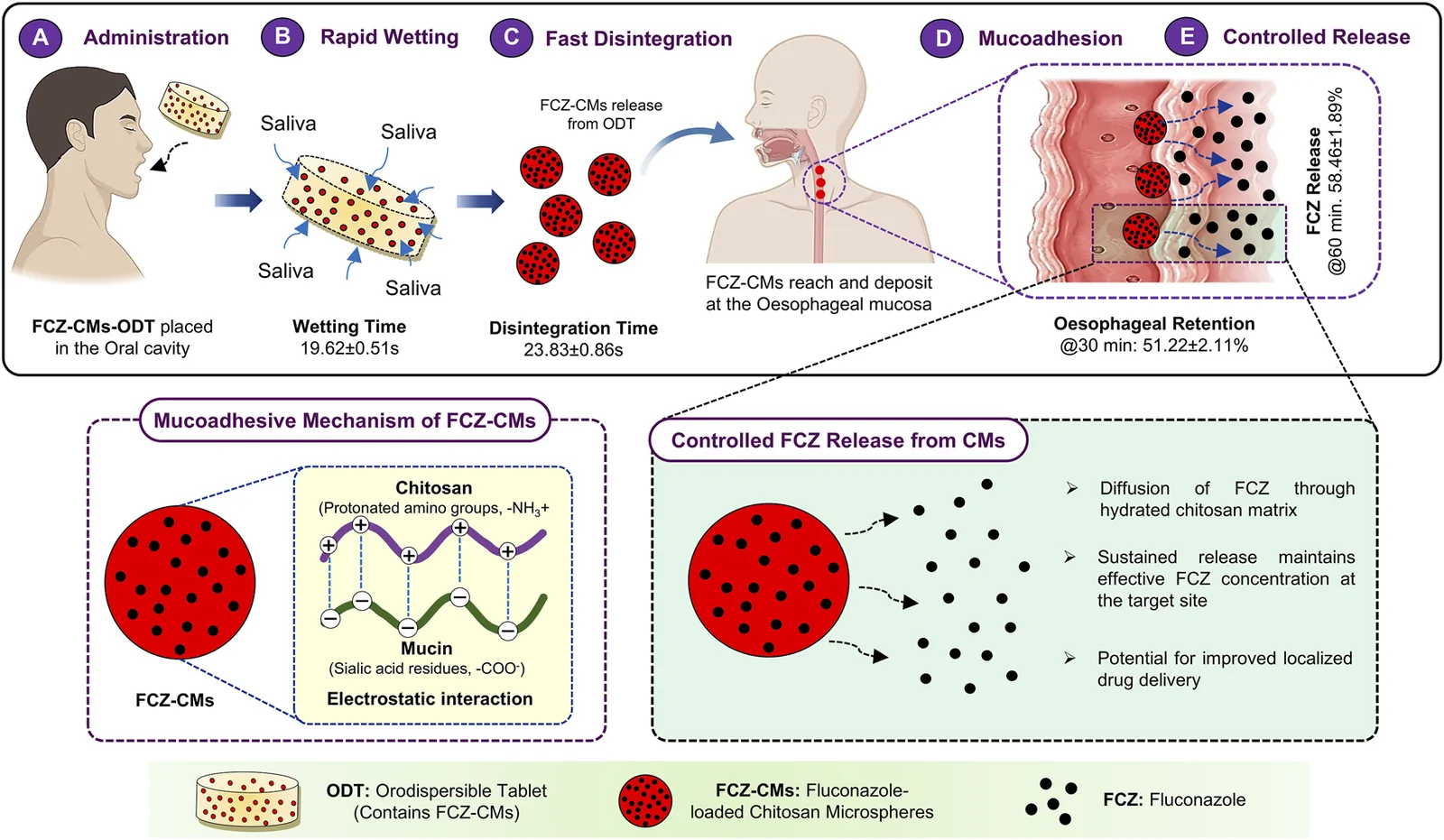
Proposed mechanism of oesophageal targeting, mucoadhesion, and controlled fluconazole release from fluconazole-loaded chitosan microsphere-based orodispersible tablets (FCZ-CMs-ODT). The schematic illustrates **(A)** administration of the ODT **(B)** rapid wetting **(C)** fast disintegration with release of FCZ-loaded chitosan microspheres **(D)** proposed oesophageal deposition and chitosan–mucin-mediated mucoadhesive retention, and **(E)** controlled diffusion of fluconazole from the chitosan microspheres. The proposed oesophageal targeting and mucoadhesive mechanism should be interpreted as a conceptual representation based on the *in vitro* and *ex vivo* findings.

### 3.5 Stability study

The stability of the FCZ-CMs-loaded ODTs was evaluated under real-time (25 ° C ± 2 ° C/60% ± 5% RH) and accelerated (40 ° C ± 2 ° C/75% ± 5% RH) storage conditions for 3 months. The results are presented in Table 11. Throughout the study period, no substantial changes in the evaluated physicochemical parameters were observed over the three-month study period.

**TABLE 11:** Stability evaluation of the orodispersible tablet containing fluconazole-loaded chitosan microspheres under real-time (25 °C ± 2 °C/60% ± 5% RH) and accelerated (40 °C ± 2 °C/75% ± 5% RH) storage conditions.

Parameters	0 Month		1 Month		2 Month		3 Month	
	Real time	Accelerated	Real time	Accelerated	Real time	Accelerated	Real time	Accelerated
DC^a^ (%)	99.14 ± 0.58	99.14 ± 0.58	98.96 ± 0.61	98.82 ± 0.65	98.74 ± 0.56	98.51 ± 0.69	98.43 ± 0.63	98.18 ± 0.72
WV^b^ (mg)	160.2 ± 0.30	160.2 ± 0.30	160.18 ± 0.34	160.11 ± 0.37	160.14 ± 0.28	160.03 ± 0.35	160.08 ± 0.32	159.97 ± 0.41
Friability (%)	0.36 ± 0.03	0.36 ± 0.03	0.37 ± 0.03	0.38 ± 0.04	0.38 ± 0.03	0.39 ± 0.03	0.39 ± 0.04	0.41 ± 0.004
Hardness (kg/cm^2^)	3.42 ± 0.15	3.42 ± 0.15	3.40 ± 0.14	3.38 ± 0.16	3.39 ± 0.16	3.35 ± 0.17	3.37 ± 0.15	3.33 ± 0.18
WT^c^ (s)	19.62 ± 0.51	19.62 ± 0.51	19.81 ± 0.54	20.04 ± 0.58	20.12 ± 0.49	20.38 ± 0.61	20.36 ± 0.56	20.71 ± 0.67
DT^d^ (s)	23.83 ± 0.86	23.83 ± 0.86	24.06 ± 0.81	24.31 ± 0.88	24.42 ± 0.85	24.78 ± 0.93	24.81 ± 0.91	25.23 ± 0.96

^a^ Drug Content.

^b^ Weight variation.

^c^ Wetting Time, and.

^d^ Disintegration Time. All values in the Table are represented as Mean ± SD (n = 3).

DC remained within the acceptable pharmacopeial limits (>98%), indicating negligible degradation of FCZ under both storage conditions. Similarly, tablet weight variation, friability, and hardness showed only marginal changes during storage, confirming that the tablet retained its mechanical strength and structural integrity. The WT and DT exhibited only slight increases under accelerated storage conditions; however, these variations were minimal and did not compromise the rapid disintegration characteristics of the formulation. The observed increase may be attributed to minor moisture-induced changes in the matrix during storage, which did not significantly affect the functionality of the dosage form. Importantly, friability remained well below the pharmacopeial acceptance limit of 1%, indicating that the tablets maintained adequate resistance to mechanical stress throughout the storage period. Overall, the absence of significant changes in physicochemical properties demonstrates that the optimised orodispersible tablet successfully preserved the stability of the incorporated FCZ-CMs under both real-time and accelerated conditions. These findings suggest that the developed formulation possesses satisfactory storage stability and is suitable for further pharmaceutical development and potential clinical translation as a localized oesophageal drug delivery system.

### 3.6 Limitations and future perspectives

Despite the promising formulation findings, several limitations should be acknowledged. First, the present study was restricted to *in vitro* and *ex vivo* evaluations, and therefore the actual oesophageal residence time and therapeutic efficacy of the formulation could not be established. Although the relatively static goat oesophageal *ex vivo* model provided a useful in sight of mucoadhesion, it cannot fully reproduce human oesophageal physiology, including peristaltic activity, salivary flow, mucus turnover, swallowing dynamics, and mucosal clearance. Furthermore, the flow rate and shear stress generated by the modified USP disintegration apparatus were not directly compared with physiological oesophageal conditions. In this context, the mechanical challenge test applied in the present model should be regarded as a standardized comparative condition rather than a direct simulation of the *in vivo* oesophageal environment.

Secondly, antifungal efficacy was not evaluated in the present study; therefore, the relationship between the observed drug-release profile and local antifungal activity remains to be established. Accordingly, the observed release of 58.46% ± 1.89% from the optimised FCZ-CMs and 53.16% ± 1.24% from the corresponding ODT at 60 min cannot be directly interpreted as therapeutic or sub-therapeutic oesophageal drug concentrations, as local mucosal drug concentrations and pharmacodynamic outcomes were not determined. Although the observed oesophageal retention would prolong local exposure, the relationship between drug release, mucosal drug concentration, and antifungal efficacy remains to be established.

Thirdly, the present data do not permit prediction of *in vivo* tissue deposition following oesophageal administration. Finally, only three-month real-time and accelerated stability studies were performed, and long-term stability and scale-up feasibility remain to be established. Future studies can therefore include *in vivo* imaging to quantify oesophageal residence and clearance, antifungal efficacy evaluation against relevant *Candida* strains, pharmacokinetic assessment, longer-term stability studies, and investigation of manufacturing scalability. These studies will be important for establishing the clinical relevance and translational potential of the proposed oesophageal-targeted delivery platform.

## 4 Summary and conclusion

The present investigation successfully demonstrated the development, optimization, and incorporation of fluconazole-loaded chitosan microspheres into an orodispersible tablet for localized oesophageal drug delivery. A systematic Design of Experiments approach enabled the identification of critical formulation variables governing particle size, entrapment efficiency, drug release, and oesophageal retention, leading to the development of an optimised microsphere formulation with desirable physicochemical characteristics and excellent mucoadhesive performance. Solid-state characterization confirmed successful incorporation of fluconazole within the chitosan matrix without evidence of drug–polymer incompatibility, while the optimised microspheres exhibited spherical morphology, high drug encapsulation, prolonged oesophageal retention, and controlled drug release.

The optimised microspheres were subsequently successfully incorporated into an orodispersible tablet using a direct compression process. The developed tablet demonstrated satisfactory mechanical strength, rapid wetting and disintegration, excellent content uniformity, and good storage stability. Importantly, comparison of dissolution profiles of optimised microspheres and the orodispersible tablet confirmed that tablet compression did not significantly alter the intrinsic release characteristics of fluconazole-loaded chitosan microspheres. These findings establish that the tablet functions as a patient-friendly carrier, rapidly releasing the microspheres while preserving their controlled-release and mucoadhesive properties. By combining rapid oral disintegration with prolonged oesophageal retention of mucoadhesive microspheres, the developed platform addresses a critical limitation of conventional oral antifungal therapy and represents a promising strategy for improving localized treatment of oesophageal candidiasis. Furthermore, the formulation strategy is scalable and amenable to commercial manufacturing, highlighting its potential for pharmaceutical development and clinical translation. Despite these promising findings, the present study was limited to *in vitro* and *ex vivo* evaluations; therefore, the actual oesophageal residence time, mucosal clearance, antifungal efficacy, and pharmacokinetic behaviour following *in vivo* administration remain to be established. Further *in vivo* studies incorporating real-time imaging, antifungal efficacy, and pharmacokinetic/pharmacodynamic evaluation are warranted to establish the therapeutic relevance and clinical applicability of this delivery platform.

## Data Availability

The raw data supporting the conclusions of this article will be made available by the authors, without undue reservation.
